# IL-17 induces AKT-dependent IL-6/JAK2/STAT3 activation and tumor progression in hepatocellular carcinoma

**DOI:** 10.1186/1476-4598-10-150

**Published:** 2011-12-15

**Authors:** Fang-Ming Gu, Quan-Lin Li, Qiang Gao, Jia-Hao Jiang, Kai Zhu, Xiao-Yong Huang, Jin-Feng Pan, Jun Yan, Jin-Hui Hu, Zheng Wang, Zhi Dai, Jia Fan, Jian Zhou

**Affiliations:** 1 Liver Cancer Institute, Zhongshan Hospital, Fudan University, Shanghai, P.R.China; 2 Endoscopy Center and Endoscopy Research Institute, Zhongshan Hospital, Fudan University, Shanghai, P.R.China; 3 Institute of Biomedical Sciences, Fudan University, Shanghai, P.R.China; 4 Department of Laboratory Medicine, Gongli Hospital, Pudong new area, Shanghai, P.R.China

## Abstract

**Background:**

The Th17 subset and IL-17 have been found in increased frequencies within certain tumors. However, their relevance in cancer biology remains controversial. This study aimed to clarify the biological action of IL-17 on hepatocellular carcinoma (HCC).

**Methods:**

Effects and underlying molecular mechanisms of IL-17 on human HCC were explored *in vitro *using exogenous IL-17 stimulation and in nude mice by implanting IL-17 overexpressed HCC cells. The clinical significance of IL-17 was investigated in tissue microarrays containing HCC tissues from 323 patients following hepatectomy using immunohistochemistry.

**Results:**

Although exogenous IL-17 showed no direct effect on the growth rate of HCC cells *in vitro*, PCR and ELISA showed that IL-17 selectively augmented the secretion of diverse proinvasive factors and transwell showed a direct promotion of invasion of HCC cells by IL-17. Furthermore, transfection of IL-17 into HCC cells significantly promoted neoangiogenesis, neutrophil recruitment and tumor growth *in vivo*. Using siRNA mediated knockdown of AKT and STAT3, we suggested that the effects of IL-17 were operated through activation of the AKT signaling in HCC, which resulted in IL-6 production. Then, IL-6 in turn activated JAK2/STAT3 signaling and subsequently up-regulated its downstream targets IL-8, MMP2, and VEGF. Supporting these findings, in human HCC tissues, immunostaining indicated that IL-17 expression was significantly and positively associated with STAT3 phosphorylation, neutrophil infiltration and increased tumor vascularity. The clinical significance of IL-17 was authenticated by revealing that the combination of intratumoral IL-17+ cells and phospho-STAT3 served as a better prognosticator for postoperative tumor recurrence than either marker alone.

**Conclusions:**

IL-17 mediated tumor-promoting role involves a direct effect on HCC cells through IL-6/JAK2/STAT3 induction by activating the AKT pathway.

## Introduction

Hepatocellular carcinoma (HCC) is the fifth most common cancer and the third most common cause of cancer-related death globally [1]. Despite advances in treatment modalities, long-term survival of HCC patients remains unsatisfactory because of the high rate of recurrence and metastasis [1]. HCC is usually secondary to inflammatory conditions due to chronic hepatitis and cirrhosis resulting from either hepatitis B/C virus infection or from non viral-related causes such as alcohol or obesity. Compelling evidence has shown that inflammation orchestrates the microenvironment around HCC, making a significant difference to cancer cell proliferation, migration, and survival [2].

T helper 17 (Th17) cells are an important inflammatory component whose main physiological role is to promote host defense against infectious agents, and are well appreciated for contributing to autoimmune diseases [3]. Recently, Th17 cells and its signature cytokine, interleukin-17 (IL-17), have been found increased frequencies within certain tumors [4-6]. However, the relationship between Th17 cells and tumor immunopathology has been controversial [7,8]. Both beneficial and detrimental direct and indirect effects of IL-17 occurred in context and tumor system dependent manners. Transfection of IL-17 into tumor cells augmented the progression of the disease in nude mice via the effects on vascular endothelium and increased neoangiogenesis [9,10]. In contrast, the same kind of experiments using syngeneic tumors in immunocompetent mice induced tumor suppression or even eradication by facilitating the recruitment of effector immune cells [11]. In clinical settings, a significant inverse correlation has been found between Th17 cell differentiation and prostate/ovarian cancer progression [4,12], and low dose cyclophosphamide has newly been shown to modulate the tumor microenvironment by decreasing Treg suppressors while favoring Th17 and Th1 cells [13]. In HCC, IL-17+ T cells have been found in increased numbers within tumors and correlate with poor survival and increased postoperative recurrence, indicating that Th17 cells and IL-17 may promote tumor progression in HCC [14]. However, the direct effects and the underlying mechanisms of IL-17 in modulating human HCC cell growth remain elusive.

Previous studies have shown that IL-17 supported tumor progression via the effects on immune cells, vascular endothelial cells and stromal cells, focusing mostly on stimulating angiogenesis and inflammation. Given that many types of tumor cells bear IL-17 receptor alpha (IL-17RA) [15,16], the specific receptor for IL-17, IL-17 may have a direct impact on the biological behavior of tumor cells in the local microenvironment. As a confirmation, in murine B16 melanoma and MB49 bladder carcinoma, IL-17 mediated tumor-promoting role involves a direct effect on tumor cells through IL-6 induction and subsequent signal transducer and activator of transcription 3 (STAT3) activation [15]. IL-6 and other members of the IL-6 family of cytokines, including IL-11, in activating the JAK-STAT3 pathway leading to cancer-promoting inflammation has been widely documented [17]. It is well-known that cytokines' role in regulating tumor progression and metastasis are highly cell-type-dependent and context-dependent, highlighting that the effects of IL-17 on HCC cells mandate specific investigation. Thus, in this study, we attempted to elucidate the exact role and associated molecular mechanism of IL-17 in HCC proliferation and invasion *in vitro *and in nude mice. The clinical relevance and prognostic significance of IL-17 in human HCC were also investigated.

## Materials and methods

### Cell lines

Two human HCC cell lines, SMMC7721 (a human HCC cell line with low metastatic potential, established by the Shanghai Institute of Cell Biology, Chinese Academy of Sciences, Shanghai, China) [18] and Huh7 (a well-differentiated and non-metastatic human HCC cell line, Japanese Cancer Research Resources Bank), were maintained in high-glucose Dulbecco's modified Eagle medium (DMEM) supplemented with 10% heat-inactivated fetal bovine serum (FBS), L-glutamine, 100 units/ml penicillin, and 100 μg/ml streptomycin. All cell lines were cultured at 37°C in a humidified incubator in 5% CO_2_.

### Quantitative reverse transcription-polymerase chain reaction (qRT-PCR)

Total RNA was extracted with Trizol Reagent (Invitrogen, Carlsbad, CA) according to the manufacturer's protocol, and reversed transcribed with RevertAid™ first-strand cDNA synthesis kit (Fermentas, Burlington, ON). IL-17 and IL-17RA mRNA levels were determined by qPCR using SYBR Premix Ex Taq (TaKaRa, Dalian, China) and normalized with β-actin using the following primers: IL-17 forward, 5'-CGC TGA TGG GAA CGT GGA CTA C-3' and reverse, 5'-GGT GGA CAA TCG GGG TGA CA-3'. IL-17R forward, 5'-GTT CAT CAC GGG CAT CTC CAT C-3' and reverse, 5'-CAG GCA GGC CAT CGG TGT ATT-3'. β-actin forward, 5'-CAA CTG GGA CGA CAT GGA GAA AAT-3' and reverse, 5'-CCA GAG GCG TAC AGG GAT AGC AC-3'. The relative gene expression was calculated with the 2^-ΔCt ^method.

### MTT, cell migration, and matrigel invasion assays

The effect of IL-17 on HCC cell growth was determined with the 3-[4,5-dimethylthiazol-2-yl]-2,5-diphenyltetrazoliumbromide (MTT) assay. Cells were seeded into 96-well flat-bottom plates (1 × 10^3^/well) and cultured for 1 to 4 days in medium supplemented with recombinant human IL-17 (0, 0.1, 0.5, 1, 5, 10, 50, 100, or 500 ng/ml; 6 wells/dose), and each experiment was repeated at least three times. After IL-17 treatment, cells were incubated with MTT (20 μl/well) at 37°C for 4 h, and then 200 μl dimethylsulfoxide was added. The absorbance of individual wells was determined at 570 nm.

The wound-healing assay was used to evaluate the ability of cell migration. Cells were grown to 80-90% confluence in 24-well plates, and a wound was made by dragging a plastic pipette tip across the cell surface. The remaining cells were washed three times to remove cell debris, and then incubated at 37°C with serum-free medium. After 48 h, migrating cells at the wound front were photographed and compared. Three separate experiments were performed.

Cell invasion assays were performed using 24-well transwells (8-μm pore size; Minipore) precoated with Matrigel (BD Biosciences, San Jose, CA). In total, 1 × 10^5 ^cells, which were suspended in 100 μl DMEM containing hIL-17 (50 ng/ml), 1% FBS, IL-6 mAb (10 ng/ml), and/or IL-6 (100 ng/ml), were added to the upper chamber, and 600 μl DMEM containing 10% FBS was placed in the lower chamber. After 36 h of incubation, Matrigel and the cells remaining in the upper chamber were removed using cotton swabs. Cells on the lower surface of the membrane were fixed in 4% paraformaldehyde and stained with Giemsa. Cells in five microscopic fields (at 200× magnification) were counted and photographed. All experiments were performed in triplicate.

Recombinant human IL-17 and IL-6, and an IL-6 neutralizing mAb (R&D Systems) were applied as appropriate.

### Immunofluorescence staining, ELISA, and western blot analysis

For immunofluorescence staining, the monoclonal antibody (mAb) IL-17RA (R&D Systems, Minneapolis, MN) was used.

The levels of IL-6, IL-1β, TNF-α, TGF-β, IL-8, G-CSF, GM-CSF, VEGF, and MMP2 in culture supernatants were measured by ELISA, following the manufacturer's instructions (R&D Systems). Intra- and inter-assay coefficients of variation were < 5% and < 10%, respectively.

Western blotting was performed as previously described [19]. The specific primary antibodies p-JAK2 (Y1007/1008), JAK2, p-STAT3 (Y705), STAT3, p-p65 NF-κB (S536), p65 NF-κB, p-AKT (S473), AKT, p-JNK (T183/Y185), JNK, p-ERK1/2 (T202/Y204), ERK1/2, p-p38 MAPK (T180/Y182) and p38 MAPK (Cell Signaling Technology, Beverly, MA), as well as MMP2 (Abcam, Cambridge, MA) were used. Glyceraldehyde-3-phosphate dehydrogenase (GAPDH; Millipore, Billerica, MA) was used as a loading control.

### Cell transfection and clone selection

STAT3- and AKT-targeted siRNAs, as well as negative control mismatch sequences, were synthesized by Shanghai GeneChem Co. using the following sense and anti-sense strands: AKT sense, GUG CCA UGA UCU GUA UUU ATT and anti-sense, UAA AUA CAG AUC AUG GCA CTT; STAT3 sense, GGG ACC UGG UGU GAA UUA UTT and anti-sense, AUA AUU CAC ACC AGG UCC CTT. Transfection into SMMC7721 and Huh7 cells was performed using Lipofectamine™ 2000 (Invitrogen) according to the manufacturer's instructions.

The pEGFP-N1-IL-17 plasmids were also constructed by Shanghai GeneChem Co. and pEGFP-N1 plasmids were used as controls. The lentiviral vector and plasmid were transfected into SMMC7721 cells as described elsewhere [20]. Stably transfected clones were validated by qRT-PCR and immunoblotting for the level of target gene expression (Additional file 1, Figure S1).

### Animal experiments

BALB/ca male nude mice (Shanghai Institute of Materia Medica, Chinese Academy of Science), 4 to 6 weeks old, were kept in laminar-flow cabinets under specific pathogen-free conditions, cared for, and handled according to the recommendations of the National Institutes of Health guidelines for care and use of laboratory animals. The experimental protocol was approved by Shanghai Medical Experimental Animal Care Committee. For the tumor challenge, 1 × 10^6 ^SMMC7721-IL-17 or SMMC7721-mock tumor cells were injected subcutaneously into nude mice, and tumor growth was monitored each week [21]. There were five animals in each group. The mice were sacrificed on day 28 after tumor implantation. The levels of IL-6, IL-8, VEGF, and MMP2 in mice serum were measured by ELISA. Tumor tissues were prepared for Western blot analysis and immunostaining. Anti-mouse CD31 and Gr-1 mAbs (Abcam) were used for immunostaining.

### Patients and follow-up

Tumor samples were obtained from 410 patients with pathologically confirmed HCC at Liver Cancer Institute of Zhongshan Hospital, Fudan University. Serial sections from 87 patients (cohort 1) treated between December 2010 and January 2011 were used for immunohistochemistry, among which 48, 21, and 18 patients were classified as TNM stage I, II, and III, respectively [22]. For tissue microarray (TMA) construction, archived tissues were obtained from 323 consecutive HCC patients treated between January 2003 and March 2004, with a median follow-up of 60 months (range, 2.0-74.0 months; cohort 2). All patients demonstrated no distant metastasis and had not received anticancer therapy before surgery. The tumor stage was determined according to the 2010 American Joint Committee on Cancer and International Union Against Cancer tumor-node-metastasis (TNM) classification system. Tumor differentiation was graded by the Edmondson grading system. The follow-up procedure was performed as described in our previous report [23]. Overall survival (OS) or time to recurrence was defined as the intervals between the dates of surgery and death or the first recurrence, respectively. Patients without death or recurrence were censored at the last follow up. Detailed clinicopathological characteristics are given in Table S1. Ethical approval was obtained from the research ethics committee of Zhongshan Hospital and written informed consent was obtained from each patient.

### Tissue microarrays and immunohistochemistry

To examine the relationship between the densities of intratumoral IL-17+ cells and CD66b+ neutrophils or microvessel densities (MVDs), 87 cases (cohort 1) were chosen for immunohistochemistry using serial whole tumor sections.

TMAs were constructed as described elsewhere [19,23] using tissues form cohort 2 (n = 323). Immunohistochemistry was conducted with the following primary mAbs: goat antihuman IL-17A (R&D Systems), mouse antihuman CD66b (BD Pharmingen, San Diego, CA) and CD34 (Abcam), and rabbit antihuman p-STAT3 (Y705) (Cell Signaling Technology). Blank controls were treated identically with primary antibodies omitted.

The capture of the photographs and measurement of positive staining density were performed as described previously [23]. Briefly, for IL-17, p-STAT3, and CD66b staining in consecutive sections from 87 HCC patients (cohort 1) and Gr-1 staining in xenografts, the sections were stained with indicated antibodies, and then were evaluated using light microscopy at 200× or 400× magnification by two investigators. Five representative fields of each case were captured. IL-17+ cell density or CD66b+ and Gr-1+ neutrophil density was evaluated according to the mean number of positive-staining cells in five count areas. For expression intensity of p-STAT3, the integrated absorbance and the area in a photograph were measured using Image-Pro Plus v6.0 software (Media Cybernetics, Inc.). A uniform setting of color segmentation was loaded for counting the integrated absorbance of all the pictures, and the mean p-STAT3 density was calculated as the product of the integrated absorbance/total area.

A Ki-67-specific monoclonal rat antibody (DAKO A/S) was used for detection and quantitation of tumor cell proliferation, while the In Situ Cell Death Detection kit-Peroxidase (Roche) was used for detection and quantitation of apoptosis. The Proliferation Index and Apoptosis Index were determined by calculating the number of Ki-67- or TUNEL-positive cells per total number of cells (hematoxylin-positive plus Ki-67- or TUNEL-positive cells) in 5 randomly selected fields at ×200, using Image-Pro Plus v6.0 software as previously described [19].

For the MVD, sections were stained with CD31 or CD34 antibody and a diaminobenzidine reaction system for immunohistochemical assessment of tumor microvessels in five randomly selected fields at 200× magnification. The average number of microvessels was calculated. For the microvessel count, any brown-stained endothelial cell or endothelial cell cluster that was clearly separated from adjacent microvessels, tumor cells, and connective elements was counted as one microvessel, irrespective of the presence of a vessel lumen [4].

IL-17 and p-STAT3 staining in TMAs were evaluated at 200× magnification using light microscopy by two investigators blinded to the clinicopathologic data of the patients. For IL-17 immunostaining, the number of positive staining cells in each 1-mm diameter cylinder were calculated manually and expressed as the mean number of the duplicates (cells/1-mm core). For the expression intensity of p-STAT3, the integrated absorbance and the area in each 1-mm diameter cylinder were measured using Image-Pro Plus v6.0 software. The mean p-STAT3 density was calculated as the product of the integrated absorbance/total area.

### Statistical analysis

Statistical analysis was performed with SPSS 16.0 software (SPSS, Chicago, IL). Measurement values were expressed as means ± standard deviations. Student's *t *test, χ^2 ^test, and Spearman's ρ correlation were used as appropriate. The cumulative recurrence and survival rates were performed by the Kaplan-Meier method (log-rank test). Cox multivariate analysis with a stepwise method (forward, likelihood ratio) was used to determine the independent prognostic factors. Two-tailed p < 0.05 was judged to be significant.

The median value of IL-17+ cell counts was used as the cutoff to dichotomize IL-17 immunostaining. The optimal cutoff for dichotomizing p-STAT3 (Y705) expression data was determined using X-tile 3.6.1 software (Yale University of New Haven) [24].

## Results

### IL-17 promotes HCC invasion and migration *in vitro*

**Figure 1:**
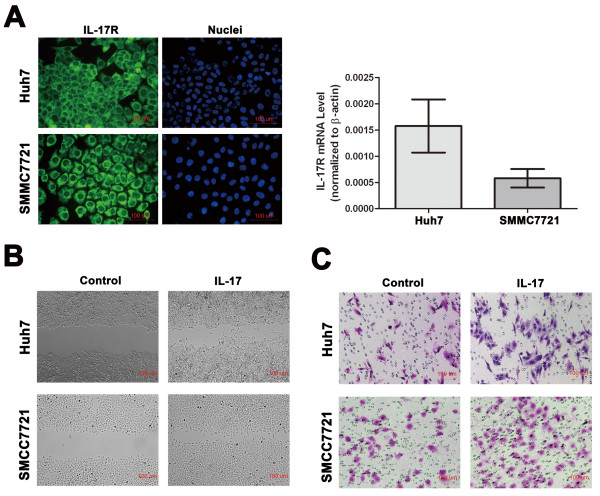
**IL-17 promotes invasion and migration of HCC *in vitro***. **(A) **As assessed by immunofluorescence staining and RT-PCR, IL-17RA expression was easily detectable in HCC cell lines (400× magnification). **(B) **Wound-healing assays revealed an evident increase in the wound closure rates of Huh7 and SMMC7721 cells after IL-17 (50 ng/ml) stimulation for 48 h (100× magnification). **(C) **Matrigel invasion assays showed that IL-17 (50 ng/ml for 36 h) significantly promoted the invasion of Huh7 and SMMC7721 cells [migration cell numbers (control vs IL-17); 37.00 ± 5.29 vs 75.67 ± 6.11, p = 0.001 for Huh7; 49.00 ± 4.00 vs 104.67 ± 13.61, p = 0.002 for SMMC7721; 200× magnification]. Each experiment was repeated at least three times. Student's *t *test was used.

The expression of IL-17RA was easily detectable by qRT-PCR and immunofluorescence in Huh7 and SMMC7721 cells (Figure 1A). Because IL-17+ T cells were significantly elevated in HCC patients and correlated with poor survival [14], we thus assumed that IL-17 could stimulate HCC cells through IL-17RA signaling pathways. Although IL-17 exerted little influence on cell proliferation (Additional file 2, Figure S2A), wound-healing assays revealed an evident increase in the wound closure rates of Huh7 and SMMC7721 cells after IL-17 (50 ng/ml) stimulation for 48 h (Figure 1B), and matrigel invasion assays showed that exogenous IL-17 (50 ng/ml for 36 h) significantly promoted the invasion of Huh7 and SMMC7721 cells (Figure 1C). These results indicated that IL-17 can promote invasion and migration of HCC *in vitro*.

### IL-17 up-regulates proinvasive factors in HCC *in vitro*

IL-17 is known for eliciting secretion of diverse inflammatory mediators in diverse cell types, including stromal cells and tumor cells. We found that IL-17 selectively up-regulated the production of IL-6, IL-8, MMP2, and VEGF in Huh7 and SMMC7721 cells (Table 1). The most prominent secretion was IL-6, with 5.8- and 6.0- fold increases, serially followed by IL-8 (2.5- and 5.6- fold), MMP2 (1.5- and 1.6- fold) and VEGF (1.2- and 1.7- fold) in Huh7 and SMMC7721 cells respectively. By contrast, in Huh7 and SMMC7721 cells, the production of IL-1β, TNF-α, TGF-β, G-CSF and GM-CSF were not significantly affected by exogenous IL-17.

**Table 1:** IL-17 affects the production of inflammatory cytokines of HCC

	Huh7 (pg/ml)			SMMC7721 (pg/ml)		
				
IL-17 (50 ng/ml)	-	+	p	-	+	p
**IL-6**	7.0 ± 1.5	40.5 ± 19.6	< 0.001	63.3 ± 33.8	380.9 ± 198.2	0.001
**IL-1β**	5.3 ± 0.4	5.53 ± 0.5	0.397	6.4 ± 1.6	5.6 ± 1.0	0.261
**TNF-α**	22.5 ± 2.3	21.3 ± 1.2	0.206	22.8 ± 3.3	26.6 ± 6.0	0.137
**TGF-β**	215.7 ± 51.9	206.5 ± 47.5	0.716	140.7 ± 83.2	143.6 ± 41.5	0.931
**IL-8**	25.4 ± 13.1	141.3 ± 133.9	0.029	208.9 ± 144.5	521.7 ± 208.3	0.004
**G-CSF**	10.9 ± 1.2	11.4 ± 2.0	0.575	20.3 ± 14.9	39.3 ± 28.1	0.112
**GM-CSF**	6.5 ± 0.5	8.3 ± 3.8	0.212	8.40 ± 2.23	15.3 ± 9.9	0.078
**VEGF**	2236.4 ± 300.3	3814.5 ± 1188.6	0.043	332.0 ± 157.7	557.8 ± 162.2	0.014
**MMP2**	327.6 ± 12.2	492.8 ± 44.5	< 0.001	351.4 ± 14.3	558.6 ± 91.2	< 0.001

ELISA-determined cytokine levels were expressed as mean ± SD. p-values were calculated by Student's *t *test.

### IL-17 activates STAT3 and AKT in HCC cells

**Figure 2:**
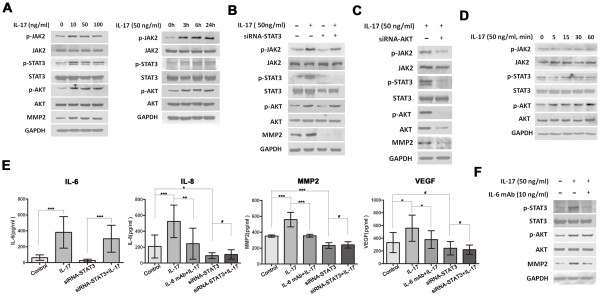
**IL-17 activates AKT and IL-6/STAT3, and up-regulates proinvasive factors production in SMMC7721 cells**. **(A) **Western blotting showed that phosphorylation of JAK2, STAT3 and AKT were obviously increased as early as 3 h after IL-17 treatment and lasted for 24 h after IL-17 stimulation. SMMC7721 cells were incubated with IL-17 at the indicated concentrations for 24 h or at 50 ng/ml for the indicated time. **(B and C) **Cells were cultured for 24 h with IL-17 (50 ng/ml). In siRNA-STAT3-SMMC7721 cells, IL-17-induced AKT and JAK2 phosphorylation were not affected, while in siRNA-AKT-SMMC7721, IL-17-induced JAK2/STAT3 phosphorylation was significantly reduced. **(D) **IL-17-induced AKT phosphorylation was obviously increased as early as 5 minutes after IL-17 treatment, while phosphorylation of JAK2 and STAT3 were not affected during the 60 minutes treatment. **(E) **HCC cells were cultured for 24 h with IL-17 (50 ng/ml) and/or IL-6 mAb (10 ng/ml), and concentrations of the proinvasive factors in culture supernatants were measured by ELISA. IL-17 selectively up-regulated the production of IL-6, IL-8, MMP2 and VEGF by tumor cells. Both IL-6 mAb and siRNA-STAT3 significantly downregulated the expression of IL-8, MMP2 and VEGF, while IL-17-induced IL-6 upregulation was not altered. **(F) **IL-6 mAb reduced STAT3 activation, whereas AKT activation by IL-17 was not affected. Data are expressed as mean ± SD; Student's *t *test; # p > 0.05; *p < 0.05, **p < 0.01, and ***p < 0.001.

Various signaling pathways are suggested to mediate IL-17 action. Here, we scrutinized the potential signaling pathways of IL-17 action in HCC cells and found that IL-17 had no effect on p38 MAPK, ERK, JNK and p65 NF-κB activation in Huh7 and SMMC7721 cells (Additional file 2, Figure S2B). Intriguingly, phosphorylation of STAT3 and AKT were obviously increased as early as 3 h after IL-17 treatment and lasted for 24 h (Figure 2A, and Additional file 3, Figure S3A). Given that expression of IL-6, IL-8, MMP2, and VEGF increased in parallel with STAT3 and AKT activation in HCC cells treated with IL-17, STAT3, and AKT signaling may play important roles in inducing these proinvasive factors and hence tumor progression.

### IL-17 promotes HCC progression via AKT-dependent IL-6/STAT3 activation

To explore the potential role of STAT3 and AKT in IL-17-mediated effects on HCC, STAT3 and AKT expression were reduced by small interfering RNA (siRNA).

First, in HCC cells exposed to STAT3-targeted siRNA (HCC-siRNA-STAT3), IL-17-induced AKT phosphorylation was not affected, while in HCC cells exposed to AKT-targeted siRNA (HCC-siRNA-AKT), IL-17-induced STAT3 phosphorylation was significantly reduced (Figure 2B and 2C, and Additional file 3, Figure S3B and S3C). Furthermore, IL-17-induced AKT phosphorylation was obviously increased as early as 5 minutes after IL-17 treatment, while phosphorylation of JAK2 and STAT3 were not affected during the 60 minutes treatment (Figure 2D). These results suggested that AKT phosphorylation was an earlier event as compared with STAT3 phosphorylation in HCC stimulated with IL-17.

Additionally, in HCC-siRNA-STAT3 cells, IL-17-induced expression of IL-8, MMP2, and VEGF were significantly inhibited, while IL-17-induced IL-6 upregulation was not affected (Figure 2B and 2E, and Additional file 3, Figure S3B and S3D). These results indicated that IL-8, MMP2, and VEGF were target genes instead of the upstream activators of STAT3. Furthermore, neutralizing IL-6 with a blocking mAb reduced JAK2 and STAT3 activation and inhibited upregulation of IL-8, MMP2, and VEGF by IL-17, whereas AKT activation by IL-17 was not affected (Figure 2E and 2F, and Additional file 3, Figure S3D and S3E). Thus, IL-6 may be the main upstream activator, rather than the downstream target of JAK2/STAT3, in the setting of IL-17 stimulation.

**Figure 3:**
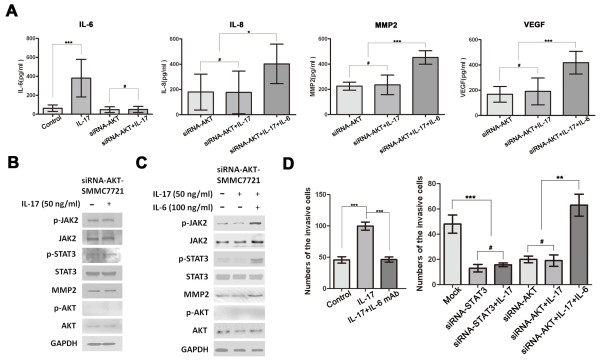
**IL-17 promotes HCC invasion via AKT-dependent IL-6/STAT3 activation**. SMMC7721 cells were exposed to AKT-targeted or STAT3-targeted siRNA, and then cultured with IL-17 (50 ng/ml) and/or IL-6 (100 ng/ml) for 24 h. **(A) **As assessed by ELISA, the expression of IL-6, IL-8, MMP2 and VEGF showed no significant change after IL-17 stimulation in siRNA-AKT-SMMC7721 cells, while expression of IL-8, MMP2 and VEGF were significantly increased after addition of IL-17 plus IL-6. **(B) **AKT-siRNA significantly downregulated JAK2/STAT3 phosphorylation induced by IL-17. **(C) **IL-6 also recovered IL-17-stimulated JAK2/STAT3 phosphorylation in siRNA-AKT-SMMC7721 cells. **(D) **As shown by Matrigel invasion assay, STAT3-siRNA significantly reversed tumor invasion by IL-17 stimulation. IL-6 mAb (10 ng/ml for 36 h) completely reversed IL-17-induced HCC invasion, while IL-6 (100 ng/ml for 36 h) completely recovered IL-17-stimulated invasion of siRNA-AKT-SMMC7721 cells. Three separate experiments were performed in each study. Data are expressed as mean ± SD; Student's *t *test; # p > 0.05; *p < 0.05, **p < 0.01, and ***p < 0.001.

By contrast, in HCC-siRNA-AKT cells, but not HCC-siRNA-STAT3 cells, IL-17-induced IL-6 upregulation was significantly blocked (Figure 2E and 3A, and Additional file 3, Figure S3D and Additional file 4, S4A). Thus, AKT siRNA both repressed IL-6 and downregulated JAK2/STAT3 phosphorylation induced by IL-17 (Figure 2C, 3A and 3B, and Additional file 3, Figure S3C, Additional file 4, S4A and S4B), indicating that IL-6/JAK2/STAT3 may be the targets of AKT in the context of IL-17. Thus, IL-17 may upregulate IL-6 production via AKT activation. IL-6 in turn activates JAK2/STAT3 and subsequently stimulates IL-8, MMP2, and VEGF production. Supporting this hypothesis, JAK2/STAT3 phosphorylation and the expression of IL-8, MMP2, and VEGF were significantly increased after addition of IL-17 plus exogenous IL-6 in siRNA-AKT-SMMC7721 and siRNA-AKT-Huh7 cells (Figure 3A and 3C, and Additional file 4, Figure S4A and S4C).

Meanwhile, STAT3-siRNA significantly reversed tumor invasion by IL-17 stimulation in HCC (Figure 3D, and Additional file 4, Figure S4D). An IL-6 neutralizing mAb reduced STAT3 activation and also completely reversed IL-17-stimulated tumor invasion *in vitro *(Figure 3D, and Additional file 4, Figure S4D), while exogenous IL-6 completely recovered IL-17-induced invasion of siRNA-AKT-SMMC7721 and siRNA-AKT-Huh7 cells (Figure 3D, and Additional file 4, Figure S4D). AKT-dependent IL-6/STAT3 activation was therefore suggested to be responsible for the tumor promoting effects of IL-17 on HCC.

### IL-17 promotes HCC progression *in vivo*

**Figure 4:**
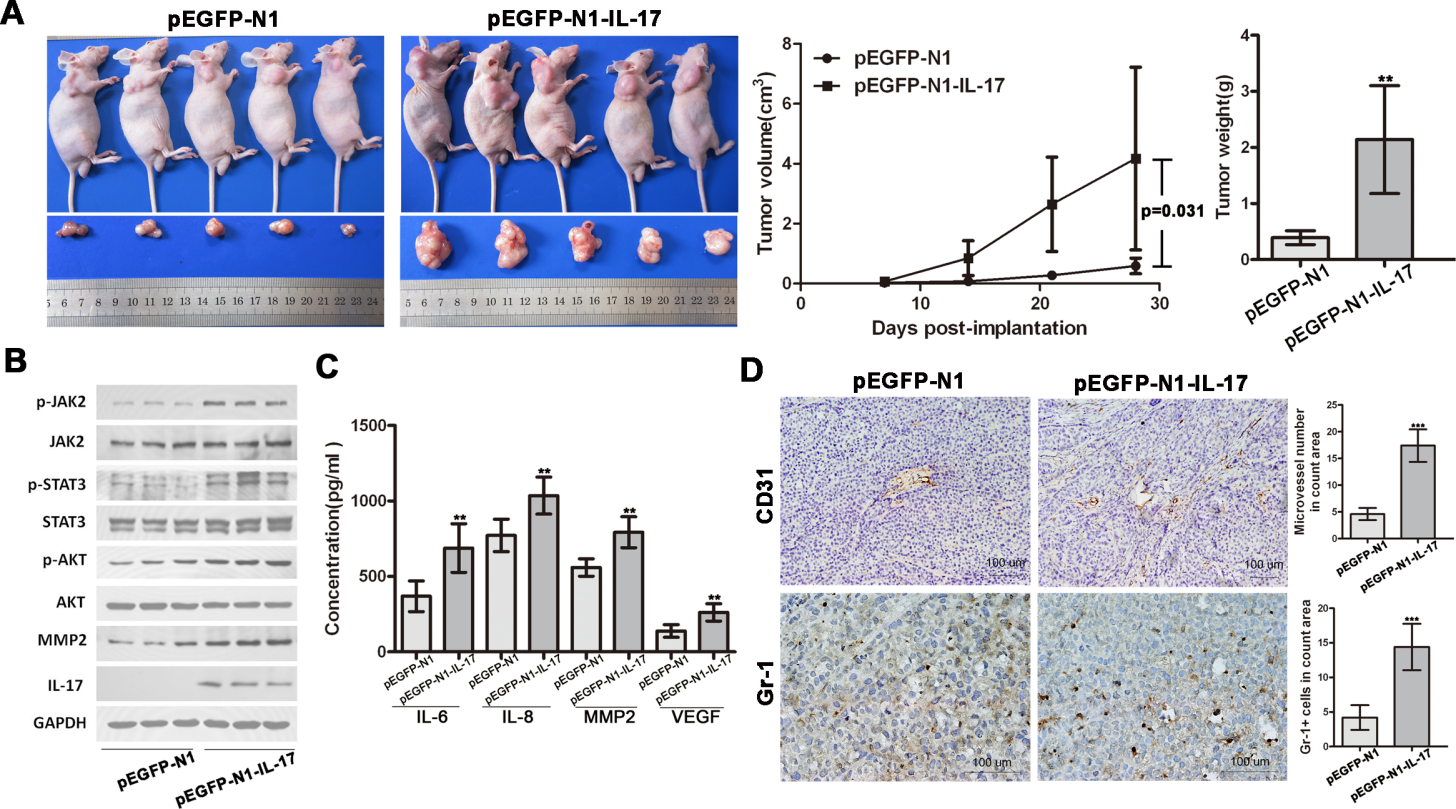
**IL-17 promotes HCC progression in nude mice**. **(A) **The tumor growth rate of SMMC7721-IL-17 was drastically increased relative to SMMC7721-mock [mean tumor volume (mock vs IL-17), 0.589 ± 0.259 vs 4.175 ± 3.050 cm^3^, p = 0.031; mean tumor weight (mock vs IL-17), 0.392 ± 0.124 vs 2.140 ± 0.963 g, p = 0.004; respectively]. **(B) **Overexpression of IL-17 promoted STAT3 and AKT activation in xenografts. **(C) **Overexpression of IL-17 markedly up-regulated production of IL-6, IL-8, MMP2 and VEGF in mice serum as measured by ELISA. **(D) **Neoangiogenesis and neutrophil recruitment in xenografts were assayed with CD31 (200× magnification) and Gr-1 staining (400× magnification). SMMC7721-IL-17-derived xenografts showed increased neoangiogenesis and neutrophil infiltration compared with the SMMC7721-mock group. Data are expressed as mean ± SD; Student's *t *test; **p < 0.01, and ***p < 0.001.

When tumor cells were injected into nude mice, the growth rate of SMMC7721-IL-17 tumors was drastically increased relative to SMMC7721-mock controls [mean tumor volume (mock vs IL-17), 0.589 ± 0.259 vs 4.175 ± 3.050 cm^3^, p = 0.031; mean tumor weight (mock vs IL-17), 0.392 ± 0.124 vs 2.140 ± 0.963 g, p = 0.004; respectively; Figure 4A]. Moreover, SMMC7721-IL-17-derived xenografts showed increased tumor cell proliferation while reduced apoptosis of HCC cells compared with the SMMC7721-mock group [proliferation index (mock vs IL-17), 71.260 ± 9.584 vs 83.820 ± 6.549, p = 0.042; apoptosis index (mock vs IL-17), 1.070 ± 0.222 vs 0.626 ± 0.320, p = 0.034; respectively; Additional file 5, Figure S5].

Increased phosphorylation of AKT was evident and the plasma concentration of IL-6 was significantly up-regulated in IL-17-transfected SMMC7721 mice compared with those in controls (Figure 4B and 4C). Overexpression of IL-17 also promoted JAK2/STAT3 activation and markedly up-regulated production of IL-8, MMP2, and VEGF (Figure 4B and 4C).

Apart from direct effects on tumor cells, MMP2 and VEGF are known for fostering angiogenesis and IL-8 is a major chemotactic factor for neutrophil recruitment. Supporting this, SMMC7721-IL-17-derived xenografts presented with more neoangiogenesis (CD31+) and Gr-1+ neutrophil infiltration than the SMMC7721-mock group (p < 0.001 for both; Figure 4D).

### Intratumoral IL-17+ cells positively correlate with p-STAT3 intensity, neutrophil and microvessel densities

**Figure 5:**
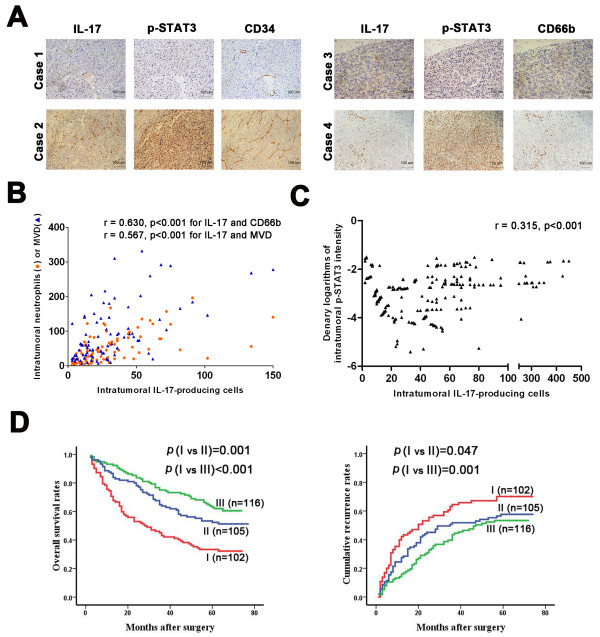
**Clinical relevance and prognostic value of IL-17+ cells**. **(A and B) **Serial whole tumor sections from 87 HCC patients were used and representative cases of immunostaining of IL-17, p-STAT3, CD34 (MVD) and CD66b (neutrophils) are shown (200× magnification). Significant positive correlations were found between the intratumoral IL-17+ cell density and MVD, as well as between the densities of intratumoral neutrophils and IL-17+ cells. **(C and D) **323 HCC patients contained in TMAs were classified into three groups: group I, high expression of both IL-17+ cells and p-STAT3; group II, high expression of one of the two markers; and group III, low expression of both markers. The intratumoral IL-17+ cell density significantly was correlated with p-STAT3 expression in this cohort as well. The 1-, 3-, and 5-year survival and cumulative recurrence rates in group I were significantly lower than those in groups II and III. Correlations between immunostaining parameters were analyzed by Spearman's rho coefficient test. The cumulative recurrence and survival rates were determined by the Kaplan-Meier method (log-rank test).

Because *in vivo *SMMC7721-IL-17 tumors were characterized by increased angiogenesis and enriched neutrophil infiltration, we further explored whether a similar phenomenon exists in human HCC tissues. By staining consecutive sections in 87 HCC patients (Figure 5A), a correlation analysis revealed that significant positive correlations were found between the density of intratumoral IL-17-producing cells and MVD (r = 0.567; p < 0.001), as well as between the densities of intratumoral CD66b+ neutrophils and IL-17+ cells (r = 0.630; p < 0.001; Figure 5B). Additionally, the p-STAT3 intensity significantly and positively correlated with levels of intratumoral IL-17+ cells, CD66b+ neutrophils, and MVD (r = 0.324 and p = 0.002; r = 0.350 and p < 0.001; r = 0.544 and p < 0.001; respectively; Additional file 6, Figure S6). Thus, IL-17+ cells may promote neoangiogenesis and neutrophil recruitment partly via STAT3 activation in human HCC.

### The combination of IL-17 and p-STAT3 is a better prognostic marker

We further investigated the prognostic value of IL-17 and its target, p-STAT3, both separately and in combination, in TMA containing 323 HCC patients (Additional file 7, Figure S7). Similarly, the density of intratumoral IL-17+ cells significantly correlated with p-STAT3 expression in this cohort (r = 0.315; p < 0.001; Figure 5C). Both IL-17^high ^and p-STAT3^high ^were significantly correlated with microvascular invasion (p = 0.016 and p = 0.013, respectively; Additional file 8, Table S1).

Among the 323 patients, the 5-year OS and cumulative recurrence rates were 50.7% and 59.7%, respectively. Univariate analysis revealed that intratumoral IL-17+ cells and p-STAT3 expression were significantly associated with recurrence and survival (Table 2). Furthermore, patients were classified into three groups: group I, high expression of both markers; group II, high expression of either markers; and group III, low expression of both markers. Significant differences in recurrence and survival were detected among the three groups (Figure 5D).

**Table 2:** Univariate and multivariate analyses of factors associated with survival and recurrence

	Overall survival			Cumulative recurrence		
	
		Multivariate			Multivariate	
				
Variables	Univariate: P	**HR **(**95%CI)**	P	Univariate: P	HR (95%CI)	P
**Age, years (≤ 51 vs > 51)**	NS	NA	NA	NS	NA	NA
**Gender (female vs male)**	0.032	NA	NS	0.013	1.765 (1.078-2.891)	0.024
**HBsAg (no vs yes)**	NS	NA	NA	NS	NA	NA
**Liver cirrhosis (no vs yes)**	NS	NA	NA	NS	NA	NA
**α-Fetoprotein (≤ 20 vs > 20 ng/mL)**	0.016	NA	NS	NS	NA	NA
**Tumor diameter (≤ 5 vs > 5 cm)**	< 0.001	2.192 (1.583-3.035)	< 0.001	< 0.001	1.613 (1.183-2.200)	0.003
**Tumor number (single vs multiple)**	0.037	NA	NS	0.136	NA	NS
**Tumor encapsulation (none vs complete)**	0.001	1.478 (1.073-2.034)	0.017	0.001	1.532 (1.126-2.084)	0.007
**Tumor differentiation (I/II vs III/IV)**	0.009	1.581 (1.118-2.237)	0.010	NS	NA	NA
**Vascular invasion (no vs yes)**	< 0.001	1.965 (1.413-2.732)	< 0.001	< 0.001	1.591 (1.157-2.188)	0.004
**TNM stage (I vs II/III)**	< 0.001	NA	NA	< 0.001	NA	NA
**IL-17 ^low ^vs IL-17 ^high^**	0.001	NA	NS	0.018	NA	NS
**p-STAT3 ^low ^vs p-STAT3 ^high^**	< 0.001	NA	NS	0.011	NA	NS
Combination of IL-17 and p-STAT3*						
**Overall**	< 0.001	NA	< 0.001	< 0.001	NA	0.009
**I vs II**	0.001	NA	NS	NS	NA	NS
**I vs III**	< 0.001	2.244 (1.529-3.293)	< 0.001	0.001	1.718 (1.193-2.473)	0.004

Note: Univariate analysis was calculated using the Kaplan-Meier method (the log-rank test). Multivariate analysis was performed using the Cox multivariate proportional hazard regression model with a stepwise method (forward, likelihood ratio).

Abbreviations: HR, hazard ratio; CI, confidence interval; HBsAg, hepatitis B surface antigen; NA, not assessed; NS, not significant; TNM, tumor-node-metastasis.

*I, IL17 ^high ^and p-STAT3 ^high^; II, IL17 ^high ^p-STAT3 ^low ^or IL17 ^low ^p-STAT3 ^high^; III, IL17^low ^and p-STAT3 ^low^_._

Significant clinicopathologic features identified in univariate analysis were adopted as covariates in multivariate Cox models. Multivariate analyses showed that IL-17+ cell and p-STAT3 expression independently correlated with OS and recurrence, irrespective of being used alone or in combination (Additional file 9, Table S2). However, concomitant high of IL-17 and p-STAT3 was superior to either marker alone in terms of hazard ratios and p values for both OS and recurrence (Additional file 9, Table S2). In multivariate analysis where IL-17+ cells, p-STAT3 expression and their combination were simultaneously adopted as covariates (Table 2), only their combination remained statistically significant. Specifically, compared with group III, hazard ratios of group I were 2.244 (95% CI, 1.529-3.293; p < 0.001) for death and 1.718 (95% CI, 1.193-2.473; p = 0.004) for recurrence (Table 2), respectively.

## Discussion

IL-17 has been shown to be elevated in several types of cancer, but how it might contribute to tumor growth is still unclear. Here, we found that IL-17 selectively augmented the secretion of various proinvasive factors and directly promoted *in vitro *invasion of HCC. Furthermore, transfection of IL-17 into HCC cells significantly promoted neoangiogenesis, neutrophil recruitment and tumor growth *in vivo*. These effects of IL-17 were suggested to be operated through activation of the AKT signaling, which resulted in IL-6 production. Then, IL-6 in turn activated JAK2/STAT3 signaling and subsequently up-regulated its downstream targets IL-8, MMP2, and VEGF. Supporting these findings, in human HCC tissues, IL-17 expression was significantly and positively associated with STAT3 phosphorylation, neutrophil infiltration, and increased tumor vascularity. Our findings thus support the notion that Th17 responses and IL-17 can promote HCC progression.

Previous studies have shown that IL-17 has diverse effects on inflammatory cells and stromal cells, focusing mostly on stimulating angiogenesis and inflammation [9-11]. Recently, in primary hepatocytes, IL-17 was shown to up-regulate a group of inflammatory cytokine genes, most of which are NF-κB target genes [25]. Given that the expression of IL-17RA was considerable in HCC cells, we thus hypothesize that IL-17-mediated tumor-promoting role involved a direct effect on HCC cells. Supporting this, we found that exogenous IL-17 directly promoted HCC cell invasion *in vitro *and enhances tumor progression *in vivo*. Although consistent with several recent publications regarding the role of IL-17 in promoting tumor growth, these findings contradict other reports suggesting that IL-17 can provide an antitumor effect against certain tumors [4,11]

The means by which IL-17 achieves its effects, either for the benefit or the detriment of the host, are largely due to the induction of new gene expression. However, different cell types appear to respond differently to IL-17 in terms of target gene expression. More recently, IL-17 has been also reported to mediate the release of proinflammatory factors and chemokines from tumor cells, including renal, lung and cervical cancers [10,26,27]. Here, we showed that IL-17 selectively up-regulated the production of proinvasive factors in HCC cells, including IL-6, IL-8, MMP2, and VEGF. As master regulators of cell dissemination, proliferation and angiogenesis, the production of these factors from HCC cells inevitably resulted in the progression of HCC. By contrast, although GM-CSF, G-CSF, TNF-α, TGF-β and IL-1β have been reported to be affected by IL-17 in other cell types [28], production of these cytokines in HCC cells was not significantly altered under IL-17.

IL-17 mediates signaling through distinct pathways in numerous inflammatory cells and tumor cells, such as MAPK, NF-κB, and STAT3 pathways [16]. We found that IL-17 significantly induced STAT3 and AKT phosphorylation, but had no obvious effect on p38 MAPK, ERK, JNK and p65 NF-κB activation in HCC cells. These indicated that STAT3 and AKT activation play an important role in the induction of proinvasive factors and hence tumor progression. Previously, IL-17 has been reported to stimulate production of IL-6 and STAT3 activation in inflammatory cells and fibroblasts in an autoimmune disease [29], as well as in cancer cells [15,30]. Furthermore, IL-6 mediated activation of STAT3 in tumor cells results in increases in anti-apoptotic, pro-proliferation, and pro-angiogenic genes [31]. In this study, using siRNA-mediated AKT and STAT3 knockdown, our data further extended these findings by indicating that IL-17 stimulated IL-6 induction was attributed to the activation of the AKT pathway; IL-6 in turn activated JAK2/STAT3 and up-regulated the transcription of the proinvasive genes. Our results were consistent with the hypothesis that, under certain conditions, AKT signaling may be the new proximal signaling mediator used by the IL-17R family to mediate downstream events [16,32,33]. To the best of our knowledge, this study is the first to demonstrate that IL-17-induced IL-6/STAT3 activation was dependent on the activation of the AKT signaling pathway in tumor cells.

Because previous studies suggested the opposite role of IL-17 in tumor systems in nude mice versus immunocompetent mice [9-11], we further verified whether our finding in the xenograft nude mouse HCC model could be reproducible in human HCC. We found that intratumoral IL-17-producing cells were positively and significantly correlated with intratumoral neutrophils and MVD, not only in transplanted HCC model but also in human HCC tissues. The results supported that aside from direct promotion effects on tumor invasion and migration, IL-17 could also promote tumor angiogenesis and neutrophil recruitment through induction of HCC cells to releasing angiogenic and chemotactic factors such as IL-8, MMPs and VEGF. Alternatively, IL-17 may directly affect vascular endothelial cells and recruite circulating neutrophils by serving as neutrophil chemotactic and angiogenic factors, which were correlated with poor prognosis in HCC [34,35]. Furthermore, the levels of IL-17+ cells positively correlated with the expression of p-STAT3, both of which were associated with the presence of microvascular invasion, and poor survival in HCC patients. The combination of intratumoral IL-17+ cells and p-STAT3 expression served as a better predictor than either used alone in HCC patients.

## Conclusions

In conclusion, our results suggested that the IL-17 mediated tumor-promoting role involved a direct effect on tumor cells through IL-6 induction by activating the AKT pathway; IL-6 in turn activated JAK2/STAT3 and up-regulated proinvasive factors IL-8, MMP2, and VEGF both *in vitro *and *in vivo*. Therapies that target IL-17 and STAT3 may be developed as potential therapeutic approaches to inhibit HCC.

## Competing interests

The authors declare that they have no competing interests.

## Authors' contributions

JZ conceived and supervised the study. FG, QL, QG performed *in vivo *and *in vitro *experiments, imaging studies and data analysis. FG, QL, QG, JJ, KZ collected clinical data. JH, ZW, ZD, JF assisted with *in vivo *and *in vitro *studies. FG, QL, QG drafted the manuscript. All authors read, edited and approved the manuscript.

## Supplementary Material

Additional file 1 **Figure S1 SMMC7721 cells are stably transfected with lentiviral-mediated pEGFP-N1-IL-17 plasmids**. The pEGFP-N1-IL-17 plasmids were constructed and pEGFP-N1 plasmids were used as controls. The lentiviral vector and plasmid were transfected into SMMC7721 cells. SMMC7721 cells were successfully transfected with pEGFP-N1-IL-17 plasmids validated by fluorescent imaging **(A)**, qRT-PCR, and immunoblotting **(B) **for the level of IL-17 expression. Click here for file

Additional file 2 **Figure S2 IL-17 shows no effect on tumor proliferation, as well as p38 MAPK, ERK, JNK, and p65 NF-κB activation in vitro**. **(A) **IL-17 had no effect on tumor proliferation as assessed by the MTT assay. Cells were cultured for 1 to 4 days in medium supplemented with IL-17 (0, 0.1, 0.5, 1, 5, 10, 50, 100 or 500 ng/ml). **(B) **Huh7 and SMMC7721 cells were incubated with IL-17 (50 ng/ml) for the indicated time. As assessed by Western blot analysis, IL-17 showed no effect on p38 MAPK, ERK, JNK, and p65 NF-κB activation. Click here for file

Additional file 3 **Figure S3 IL-17 activates AKT and IL-6/STAT3, and up-regulates proinvasive factors production in Huh7 cells**. **(A) **Western blotting showed that phosphorylation of JAK2, STAT3 and AKT were obviously increased as early as 3 h after IL-17 treatment and lasted for 24 h after IL-17 stimulation. Huh7 cells were incubated with IL-17 at the indicated concentrations for 24 h or at 50 ng/ml for the indicated time. **(B and C) **Cells were cultured for 24 h with IL-17 (50 ng/ml). In siRNA-STAT3-Huh7 cells, IL-17-induced AKT and JAK2 phosphorylation were not affected, while in siRNA-AKT- Huh7, IL-17-induced JAK2/STAT3 phosphorylation was significantly reduced. **(D) **HCC cells were cultured for 24 h with IL-17 (50 ng/ml) and/or IL-6 mAb (10 ng/ml), and concentrations of the proinvasive factors in culture supernatants were measured by ELISA. IL-17 selectively up-regulated the production of IL-6, IL-8, MMP2 and VEGF by tumor cells. Both IL-6 mAb and siRNA-STAT3 significantly downregulated the expression of IL-8, MMP2 and VEGF, while IL-17-induced IL-6 upregulation was not altered. **(E) **IL-6 mAb reduced STAT3 activation, whereas AKT activation by IL-17 was not affected. Click here for file

Additional file 4 **Figure S4 IL-17 promotes HCC invasion via AKT-dependent IL-6/STAT3 activation**. Huh7 cells were exposed to AKT-targeted or STAT3-targeted siRNA, and then cultured with IL-17 (50 ng/ml) and/or IL-6 (100 ng/ml) for 24 h. **(A) **As assessed by ELISA, the expression of IL-6, IL-8, MMP2 and VEGF showed no significant change after IL-17 stimulation in siRNA-AKT-Huh7 cells, while expression of IL-8, MMP2 and VEGF were significantly increased after addition of IL-17 plus IL-6. **(B) **AKT-siRNA significantly downregulated JAK2/STAT3 phosphorylation induced by IL-17. **(C) **IL-6 also recovered IL-17-stimulated JAK2/STAT3 phosphorylation in siRNA-AKT-Huh7 cells. **(D) **As shown by Matrigel invasion assay, STAT3-siRNA significantly reversed tumor invasion by IL-17 stimulation. IL-6 mAb (10 ng/ml for 36 h) completely reversed IL-17-induced HCC invasion, while IL-6 (100 ng/ml for 36 h) completely recovered IL-17-stimulated invasion of siRNA-AKT-Huh7 cells. Three separate experiments were performed in each study. Click here for file

Additional file 5 **Figure S5 IL-17 promotes tumor cell proliferation and inhibites apoptosis of HCC cells in nude mice**. Proliferation index and apoptosis index in xenografts were assayed with Ki-67 and TUNEL staining (200× magnification). SMMC7721-IL-17-derived xenografts showed increased tumor cell proliferation while reduced apoptosis of HCC cells compared with the SMMC7721-mock group. Click here for file

Additional file 6 **Figure S6 The intratumoral p-STAT3 intensity is positively correlated with IL-17+ cell, neutrophil and microvessel densities**. Serial whole tumor sections from 87 HCC patients were used for immunostaining. The intratumoral p-STAT3 staining intensity was significantly and positively correlated with the levels of intratumoral IL-17+ cells, neutrophils, and MVD. Correlations between immunostaining parameters were analyzed by Spearman's rho coefficient test. Click here for file

Additional file 7 **Figure S7 Representative cases of immunohistochemical staining of IL-17 and p-STAT3 in a tissue microarray**. Consecutive sections of case 1 and case 2 showed high IL-17+ cells and p-STAT3 staining, while consecutive sections of case 3 and 4 showed low IL-17+ cells and p-STAT3 staining. Click here for file

Additional file 8 **Table S1 Correlation between IL-17 or p-STAT3 and clinicopathological characteristics**. Both IL-17^high ^and p-STAT3^high ^were significantly correlated with microvascular invasion. Click here for file

Additional file 9 **Table S2 Multivariate analyses of prognosis factors associated with survival**. IL-17+ cell and p-STAT3 expression independently correlated with OS and recurrence, irrespective of being used alone or in combination. However, concomitant high of IL-17 and p-STAT3 was superior to either marker alone in terms of hazard ratios and p values for both OS and recurrence. Click here for file

## References

[B1] El-Serag HB Rudolph KL Hepatocellular carcinoma: epidemiology and molecular carcinogenesis Gastroenterology 2007 132 2557 2576 10.1053/j.gastro.2007.04.061 17570226

[B2] Ferrone C Dranoff G Dual roles for immunity in gastrointestinal cancers J Clin Oncol 2010 28 4045 4051 10.1200/JCO.2010.27.9992 20644090 PMC4872327

[B3] Miossec P Korn T Kuchroo VK Interleukin-17 and type 17 helper T cells N Engl J Med 2009 361 888 898 10.1056/NEJMra0707449 19710487

[B4] Kryczek I Banerjee M Cheng P Vatan L Szeliga W Wei S Huang E Finlayson E Simeone D Welling TH Chang A Coukos G Liu R Zou W Phenotype, distribution, generation, and functional and clinical relevance of Th17 cells in the human tumor environments Blood 2009 114 1141 1149 10.1182/blood-2009-03-208249 19470694 PMC2723011

[B5] Steiner GE Newman ME Paikl D Stix U Memaran-Dagda N Lee C Marberger MJ Expression and function of pro-inflammatory interleukin IL-17 and IL-17 receptor in normal, benign hyperplastic, and malignant prostate Prostate 2003 56 171 182 10.1002/pros.10238 12772186

[B6] Zhang B Rong G Wei H Zhang M Bi J Ma L Xue X Wei G Liu X Fang G The prevalence of Th17 cells in patients with gastric cancer Biochem Biophys Res Commun 2008 374 533 537 10.1016/j.bbrc.2008.07.060 18655770

[B7] Bronte V Th17 and cancer: friends or foes? Blood 2008 112 214 18606882 10.1182/blood-2008-04-149260

[B8] Murugaiyan G Saha B Protumor vs antitumor functions of IL-17 J Immunol 2009 183 4169 4175 10.4049/jimmunol.0901017 19767566

[B9] Numasaki M Fukushi J Ono M Narula SK Zavodny PJ Kudo T Robbins PD Tahara H Lotze MT Interleukin-17 promotes angiogenesis and tumor growth Blood 2003 101 2620 2627 10.1182/blood-2002-05-1461 12411307

[B10] Tartour E Fossiez F Joyeux I Galinha A Gey A Claret E Sastre-Garau X Couturier J Mosseri V Vives V Banchereau J Fridman WH Wijdenes J Lebecque S Sautes-Fridman C Interleukin 17, a T-cell-derived cytokine, promotes tumorigenicity of human cervical tumors in nude mice Cancer Res 1999 59 3698 3704 10446984

[B11] Benchetrit F Ciree A Vives V Warnier G Gey A Sautes-Fridman C Fossiez F Haicheur N Fridman WH Tartour E Interleukin-17 inhibits tumor cell growth by means of a T-cell-dependent mechanism Blood 2002 99 2114 2121 10.1182/blood.V99.6.2114 11877287

[B12] Sfanos KS Bruno TC Maris CH Xu L Thoburn CJ DeMarzo AM Meeker AK Isaacs WB Drake CG Phenotypic analysis of prostate-infiltrating lymphocytes reveals TH17 and Treg skewing Clin Cancer Res 2008 14 3254 3261 10.1158/1078-0432.CCR-07-5164 18519750 PMC3082357

[B13] Viaud S Flament C Zoubir M Pautier P LeCesne A Ribrag V Soria JC Marty V Vielh P Robert C Chaput N Zitvogel L Cyclophosphamide induces differentiation of Th17 cells in cancer patients Cancer Res 2011 71 661 665 10.1158/0008-5472.CAN-10-1259 21148486

[B14] Zhang JP Yan J Xu J Pang XH Chen MS Li L Wu C Li SP Zheng L Increased intratumoral IL-17-producing cells correlate with poor survival in hepatocellular carcinoma patients J Hepatol 2009 50 980 989 10.1016/j.jhep.2008.12.033 19329213

[B15] Wang L Yi T Kortylewski M Pardoll DM Zeng D Yu H IL-17 can promote tumor growth through an IL-6-Stat3 signaling pathway J Exp Med 2009 206 1457 1464 10.1084/jem.20090207 19564351 PMC2715087

[B16] Gaffen SL Structure and signalling in the IL-17 receptor family Nat Rev Immunol 2009 9 556 567 10.1038/nri2586 19575028 PMC2821718

[B17] Yu H Pardoll D Jove R STATs in cancer inflammation and immunity: a leading role for STAT3 Nat Rev Cancer 2009 9 798 809 10.1038/nrc2734 19851315 PMC4856025

[B18] Ye QH Qin LX Forgues M He P Kim JW Peng AC Simon R Li Y Robles AI Chen Y Ma ZC Wu ZQ Ye SL Liu YK Tang ZY Wang XW Predicting hepatitis B virus-positive metastatic hepatocellular carcinomas using gene expression profiling and supervised machine learning Nat Med 2003 9 416 423 10.1038/nm843 12640447

[B19] Gao Q Wang XY Qiu SJ Yamato I Sho M Nakajima Y Zhou J Li BZ Shi YH Xiao YS Xu Y Fan J Overexpression of PD-L1 significantly associates with tumor aggressiveness and postoperative recurrence in human hepatocellular carcinoma Clin Cancer Res 2009 15 971 979 10.1158/1078-0432.CCR-08-1608 19188168

[B20] Shi GM Ke AW Zhou J Wang XY Xu Y Ding ZB Devbhandari RP Huang XY Qiu SJ Shi YH Dai Z Yang XR Yang GH Fan J CD151 modulates expression of matrix metalloproteinase 9 and promotes neoangiogenesis and progression of hepatocellular carcinoma Hepatology 2010 52 183 196 20578262 10.1002/hep.23661

[B21] Fu J Chen Y Cao J Luo T Qian YW Yang W Ren YB Su B Cao GW Yang Y Yan YQ Shen F Wu MC Feng GS Wang HY p28GANK overexpression accelerates hepatocellular carcinoma invasiveness and metastasis via phosphoinositol 3-kinase/AKT/hypoxia-inducible factor-1alpha pathways Hepatology 2011 53 181 192 10.1002/hep.24015 21254169

[B22] Berasain C Castillo J Perugorria MJ Latasa MU Prieto J Avila MA Inflammation and Liver Cancer Annals of the New York Academy of Sciences 2009 1155 206 221 10.1111/j.1749-6632.2009.03704.x 19250206

[B23] Gao Q Qiu SJ Fan J Zhou J Wang XY Xiao YS Xu Y Li YW Tang ZY Intratumoral balance of regulatory and cytotoxic T cells is associated with prognosis of hepatocellular carcinoma after resection J Clin Oncol 2007 25 2586 2593 10.1200/JCO.2006.09.4565 17577038

[B24] Camp RL Dolled-Filhart M Rimm DL X-tile: a new bio-informatics tool for biomarker assessment and outcome-based cut-point optimization Clin Cancer Res 2004 10 7252 7259 10.1158/1078-0432.CCR-04-0713 15534099

[B25] Sparna T Retey J Schmich K Albrecht U Naumann K Gretz N Fischer HP Bode JG Merfort I Genome-wide comparison between IL-17 and combined TNF-alpha/IL-17 induced genes in primary murine hepatocytes BMC Genomics 2010 11 226 10.1186/1471-2164-11-226 20374638 PMC2858152

[B26] Inozume T Hanada K Wang QJ Yang JC IL-17 secreted by tumor reactive T cells induces IL-8 release by human renal cancer cells J Immunother 2009 32 109 117 10.1097/CJI.0b013e31819302da 19238009 PMC7386431

[B27] Numasaki M Watanabe M Suzuki T Takahashi H Nakamura A McAllister F Hishinuma T Goto J Lotze MT Kolls JK Sasaki H IL-17 enhances the net angiogenic activity and in vivo growth of human non-small cell lung cancer in SCID mice through promoting CXCR-2-dependent angiogenesis J Immunol 2005 175 6177 6189 16237115 10.4049/jimmunol.175.9.6177

[B28] Onishi RM Gaffen SL Interleukin-17 and its target genes: mechanisms of interleukin-17 function in disease Immunology 2011 129 311 321 10.1111/j.1365-2567.2009.03240.x PMC2826676 20409152

[B29] Ogura H Murakami M Okuyama Y Tsuruoka M Kitabayashi C Kanamoto M Nishihara M Iwakura Y Hirano T Interleukin-17 promotes autoimmunity by triggering a positive-feedback loop via interleukin-6 induction Immunity 2008 29 628 636 10.1016/j.immuni.2008.07.018 18848474

[B30] Wang L Yi T Zhang W Pardoll DM Yu H IL-17 enhances tumor development in carcinogen-induced skin cancer Cancer Res 2010 70 10112 10120 10.1158/0008-5472.CAN-10-0775 21159633 PMC3059780

[B31] Yu H Kortylewski M Pardoll D Crosstalk between cancer and immune cells: role of STAT3 in the tumour microenvironment Nat Rev Immunol 2007 7 41 51 10.1038/nri1995 17186030

[B32] Huang F Kao CY Wachi S Thai P Ryu J Wu R Requirement for both JAK-mediated PI3K signaling and ACT1/TRAF6/TAK1-dependent NF-kappaB activation by IL-17A in enhancing cytokine expression in human airway epithelial cells J Immunol 2007 179 6504 6513 17982039 10.4049/jimmunol.179.10.6504

[B33] Hwang SY Kim JY Kim KW Park MK Moon Y Kim WU Kim HY IL-17 induces production of IL-6 and IL-8 in rheumatoid arthritis synovial fibroblasts via NF-kappaB- and PI3-kinase/Akt-dependent pathways Arthritis Res Ther 2004 6 R120 128 10.1186/ar1038 15059275 PMC400429

[B34] Roussel L Houle F Chan C Yao Y Berube J Olivenstein R Martin JG Huot J Hamid Q Ferri L Rousseau S IL-17 promotes p38 MAPK-dependent endothelial activation enhancing neutrophil recruitment to sites of inflammation J Immunol 2010 184 4531 4537 10.4049/jimmunol.0903162 20228195

[B35] Li YW Qiu SJ Fan J Zhou J Gao Q Xiao YS Xu YF Intratumoral neutrophils: a poor prognostic factor for hepatocellular carcinoma following resection J Hepatol 2010 54 497 505 21112656 10.1016/j.jhep.2010.07.044

