# Supplementary material for: IL-17 induces AKT-dependent IL-6/JAK2/STAT3 activation and tumor progression in hepatocellular carcinoma
Source: Mol Cancer. 2011 Dec 15;10:150. doi: 10.1186/1476-4598-10-150 (PMC3310750; doi:10.1186/1476-4598-10-150)

Additional file 1

Figure S1 SMMC7721 cells are stably transfected with lentiviral-mediated pEGFP-N1-IL-17 plasmids. The pEGFP-N1-IL-17 plasmids were constructed and pEGFP-N1 plasmids were used as controls. The lentiviral vector and plasmid were transfected into SMMC7721 cells. SMMC7721 cells were successfully transfected with pEGFP-N1-IL-17 plasmids validated by fluorescent imaging **(A)**, qRT–PCR, and immunoblotting **(B)** for the level of IL-17 expression. Data are expressed as mean ± SD; Student's *t* test; ***p < 0.001.

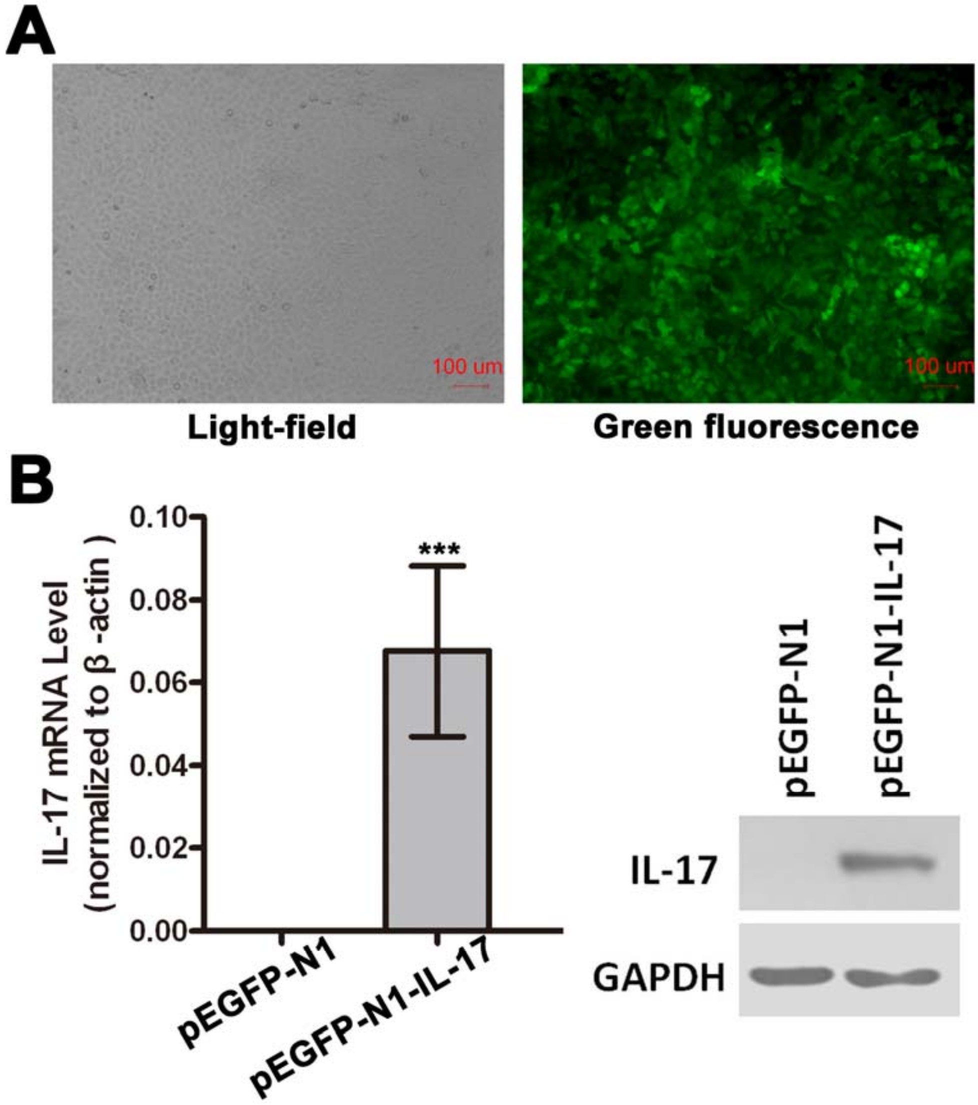

Supplement: Additional file 1 — Figure S1 SMMC7721 cells are stably transfected with lentiviral-mediated pEGFP-N1-IL-17 plasmids. The pEGFP-N1-IL-17 plasmids were constructed and pEGFP-N1 plasmids were used as controls. The lentiviral vector and plasmid were transfected into SMMC7721 cells. SMMC7721 cells were successfully transfected with pEGFP-N1-IL-17 plasmids validated by fluorescent imaging (A), qRT-PCR, and immunoblotting (B) for the level of IL-17 expression. [file 1476-4598-10-150-S1.PDF]
