# Supplementary material for: IL-17 induces AKT-dependent IL-6/JAK2/STAT3 activation and tumor progression in hepatocellular carcinoma
Source: Mol Cancer. 2011 Dec 15;10:150. doi: 10.1186/1476-4598-10-150 (PMC3310750; doi:10.1186/1476-4598-10-150)

Additional file 2

Figure S2 IL-17 shows no effect on tumor proliferation, as well as p38 MAPK, ERK, JNK, and p65 NF-κB activation in vitro. (A) IL-17 had no effect on tumor proliferation as assessed by the MTT assay. Cells were cultured for 1 to 4 days in medium supplemented with IL-17 (0, 0.1, 0.5, 1, 5, 10, 50, 100 or 500 ng/ml). **(B)** Huh7 and SMMC7721 cells were incubated with IL-17 (50 ng/ml) for the indicated time. As assessed by Western blot analysis, IL-17 showed no effect on p38 MAPK, ERK, JNK, and p65 NF-κB activation.

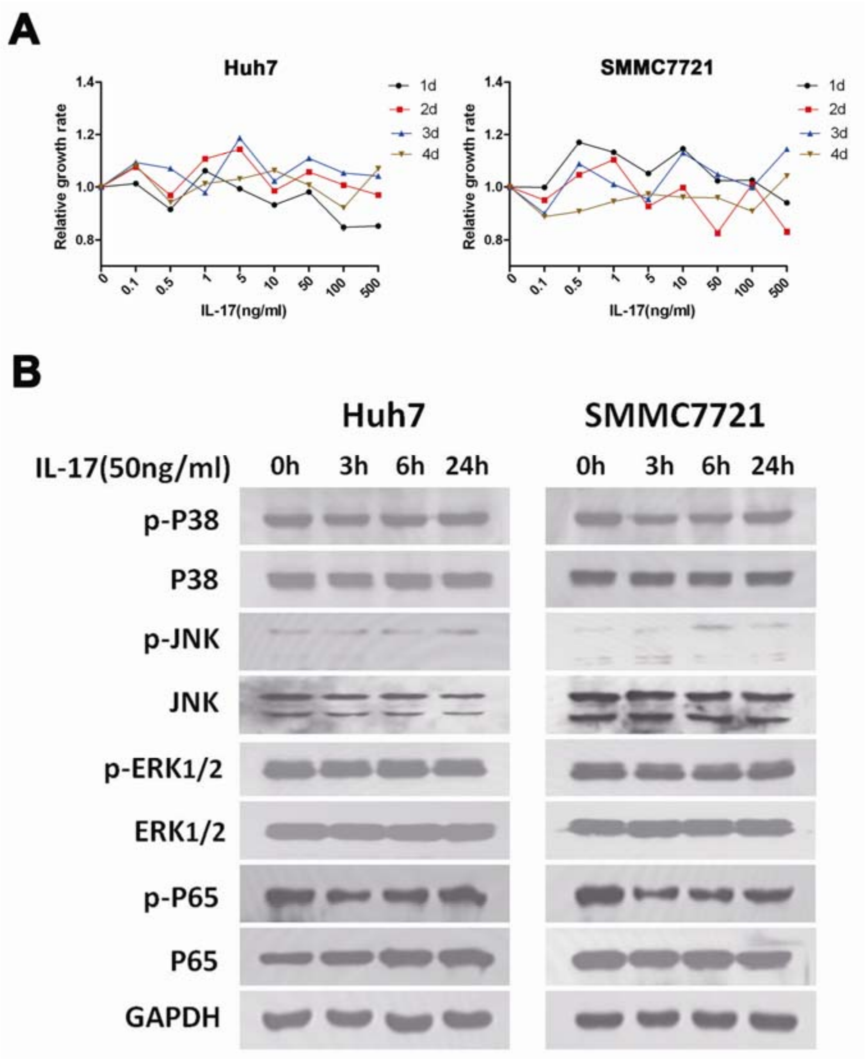

Supplement: Additional file 2 — Figure S2 IL-17 shows no effect on tumor proliferation, as well as p38 MAPK, ERK, JNK, and p65 NF-κB activation in vitro. (A) IL-17 had no effect on tumor proliferation as assessed by the MTT assay. Cells were cultured for 1 to 4 days in medium supplemented with IL-17 (0, 0.1, 0.5, 1, 5, 10, 50, 100 or 500 ng/ml). (B) Huh7 and SMMC7721 cells were incubated with IL-17 (50 ng/ml) for the indicated time. As assessed by Western blot analysis, IL-17 showed no effect on p38 MAPK, ERK, JNK, and p65 NF-κB activation. [file 1476-4598-10-150-S2.PDF]
