# Supplementary material for: IL-17 induces AKT-dependent IL-6/JAK2/STAT3 activation and tumor progression in hepatocellular carcinoma
Source: Mol Cancer. 2011 Dec 15;10:150. doi: 10.1186/1476-4598-10-150 (PMC3310750; doi:10.1186/1476-4598-10-150)
Supplement: Additional file 3 — Figure S3 IL-17 activates AKT and IL-6/STAT3, and up-regulates proinvasive factors production in Huh7 cells. (A) Western blotting showed that phosphorylation of JAK2, STAT3 and AKT were obviously increased as early as 3 h after IL-17 treatment and lasted for 24 h after IL-17 stimulation. Huh7 cells were incubated with IL-17 at the indicated concentrations for 24 h or at 50 ng/ml for the indicated time. (B and C) Cells were cultured for 24 h with IL-17 (50 ng/ml). In siRNA-STAT3-Huh7 cells, IL-17-induced AKT and JAK2 phosphorylation were not affected, while in siRNA-AKT- Huh7, IL-17-induced JAK2/STAT3 phosphorylation was significantly reduced. (D) HCC cells were cultured for 24 h with IL-17 (50 ng/ml) and/or IL-6 mAb (10 ng/ml), and concentrations of the proinvasive factors in culture supernatants were measured by ELISA. IL-17 selectively up-regulated the production of IL-6, IL-8, MMP2 and VEGF by tumor cells. Both IL-6 mAb and siRNA-STAT3 significantly downregulated the expression of IL-8, MMP2 and VEGF, while IL-17-induced IL-6 upregulation was not altered. (E) IL-6 mAb reduced STAT3 activation, whereas AKT activation by IL-17 was not affected. [file 1476-4598-10-150-S3.PDF]

Additional file 3

Figure S3 IL-17 activates AKT and IL-6/STAT3, and up-regulates proinvasive factors production in Huh7 cells. (A) Western blotting showed that phosphorylation of JAK2, STAT3 and AKT were obviously increased as early as 3 h after IL-17 treatment and lasted for 24 h after IL-17 stimulation. Huh7 cells were incubated with IL-17 at the indicated concentrations for 24 h or at 50 ng/ml for the indicated time. **(B and C)** Cells were cultured for 24 h with IL-17 (50 ng/ml). In siRNA-STAT3-Huh7 cells, IL-17-induced AKT and JAK2 phosphorylation were not affected, while in siRNA-AKT- Huh7, IL-17-induced JAK2/STAT3 phosphorylation was significantly reduced. **(D)** HCC cells were cultured for 24 h with IL-17 (50 ng/ml) and/or IL-6 mAb (10 ng/ml), and concentrations of the proinvasive factors in culture supernatants were measured by ELISA. IL-17 selectively up-regulated the production of IL-6, IL-8, MMP2 and VEGF by tumor cells. Both IL-6 mAb and siRNA-STAT3 significantly downregulated the expression of IL-8, MMP2 and VEGF, while IL-17-induced IL-6 upregulation was not altered. **(E)** IL-6 mAb reduced STAT3 activation, whereas AKT activation by IL-17 was not affected. Data are expressed as mean ± SD; Student's *t* test; # $p > 0.05$; * $p < 0.05$, ** $p < 0.01$, and *** $p < 0.001$.

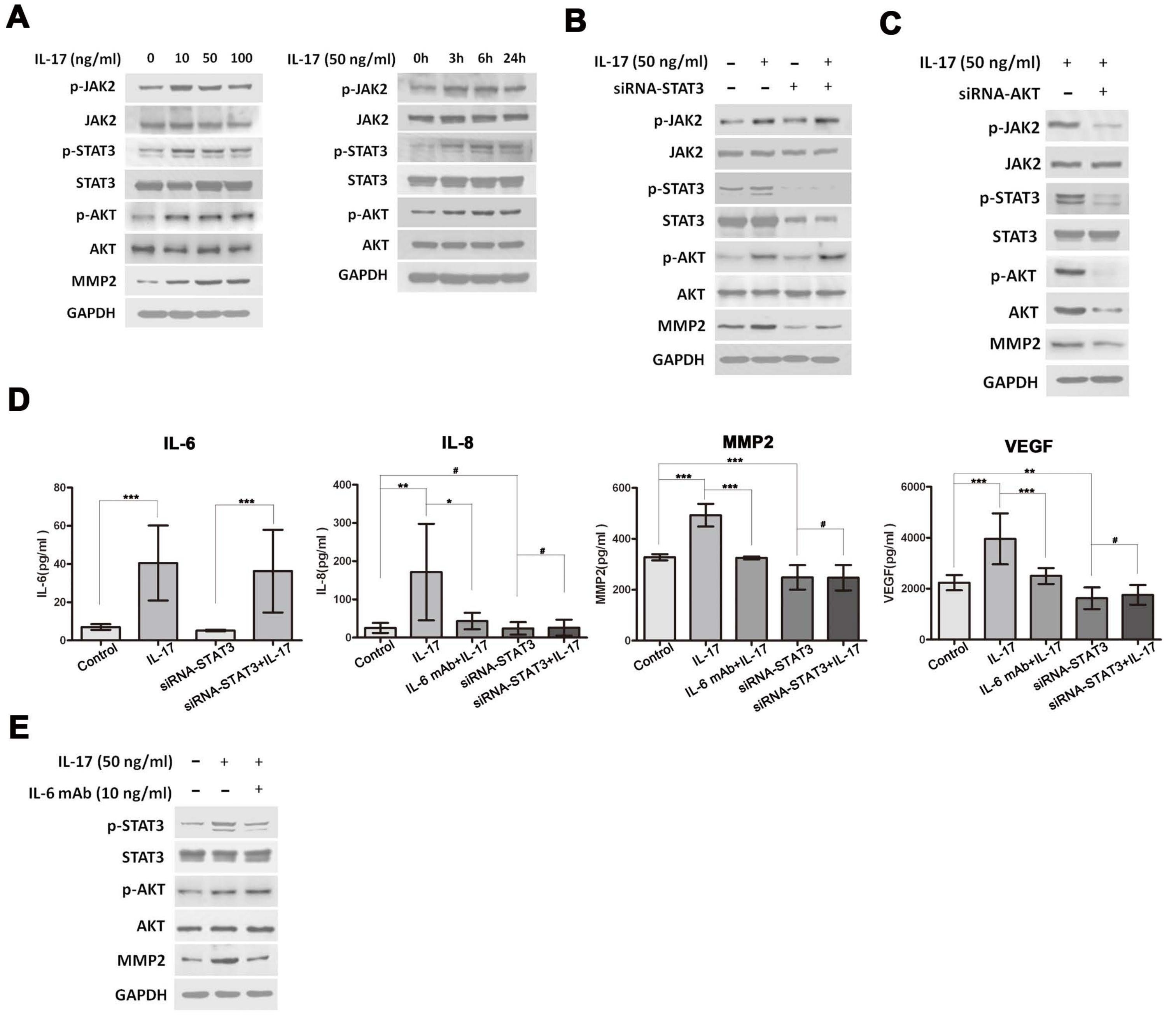
A
IL-17 (ng/ml) 0 10 50 100
p-JAK2
JAK2
p-STAT3
STAT3
p-AKT
AKT
MMP2
GAPDH
IL-17 (50 ng/ml) 0h 3h 6h 24h
p-JAK2
JAK2
p-STAT3
STAT3
p-AKT
AKT
GAPDH
B
IL-17 (50 ng/ml) − + − +
siRNA-STAT3 − − + +
p-JAK2
JAK2
p-STAT3
STAT3
p-AKT
AKT
MMP2
GAPDH
C
IL-17 (50 ng/ml) + +
siRNA-AKT − +
p-JAK2
JAK2
p-STAT3
STAT3
p-AKT
AKT
MMP2
GAPDH
D
IL-6
IL-6(pg/ml)
0
20
40
60
80
***
***
Control
IL-17
siRNA-STAT3
siRNA-STAT3+IL-17
IL-8
IL-8(pg/ml)
0
100
200
300
400
**
*
#
#
Control
IL-17
IL-6 mAb+IL-17
siRNA-STAT3
siRNA-STAT3+IL-17
MMP2
MMP2(pg/ml)
0
200
400
600
***
***
***
#
Control
IL-17
IL-6 mAb+IL-17
siRNA-STAT3
siRNA-STAT3+IL-17
VEGF
VEGF(pg/ml)
0
2000
4000
6000
***
***
**
#
Control
IL-17
IL-6 mAb+IL-17
siRNA-STAT3
siRNA-STAT3+IL-17
E
IL-17 (50 ng/ml) − + +
IL-6 mAb (10 ng/ml) − − +
p-STAT3
STAT3
p-AKT
AKT
MMP2
GAPDH
