# Supplementary material for: IL-17 induces AKT-dependent IL-6/JAK2/STAT3 activation and tumor progression in hepatocellular carcinoma
Source: Mol Cancer. 2011 Dec 15;10:150. doi: 10.1186/1476-4598-10-150 (PMC3310750; doi:10.1186/1476-4598-10-150)
Supplement: Additional file 4 — Figure S4 IL-17 promotes HCC invasion via AKT-dependent IL-6/STAT3 activation. Huh7 cells were exposed to AKT-targeted or STAT3-targeted siRNA, and then cultured with IL-17 (50 ng/ml) and/or IL-6 (100 ng/ml) for 24 h. (A) As assessed by ELISA, the expression of IL-6, IL-8, MMP2 and VEGF showed no significant change after IL-17 stimulation in siRNA-AKT-Huh7 cells, while expression of IL-8, MMP2 and VEGF were significantly increased after addition of IL-17 plus IL-6. (B) AKT-siRNA significantly downregulated JAK2/STAT3 phosphorylation induced by IL-17. (C) IL-6 also recovered IL-17-stimulated JAK2/STAT3 phosphorylation in siRNA-AKT-Huh7 cells. (D) As shown by Matrigel invasion assay, STAT3-siRNA significantly reversed tumor invasion by IL-17 stimulation. IL-6 mAb (10 ng/ml for 36 h) completely reversed IL-17-induced HCC invasion, while IL-6 (100 ng/ml for 36 h) completely recovered IL-17-stimulated invasion of siRNA-AKT-Huh7 cells. Three separate experiments were performed in each study. [file 1476-4598-10-150-S4.PDF]

Additional file 4

Figure S4 IL-17 promotes HCC invasion via AKT-dependent IL-6/STAT3 activation. Huh7 cells were exposed to AKT-targeted or STAT3-targeted siRNA, and then cultured with IL-17 (50 ng/ml) and/or IL-6 (100 ng/ml) for 24 h. **(A)** As assessed by ELISA, the expression of IL-6, IL-8, MMP2 and VEGF showed no significant change after IL-17 stimulation in siRNA-AKT-Huh7 cells, while expression of IL-8, MMP2 and VEGF were significantly increased after addition of IL-17 plus IL-6. **(B)** AKT-siRNA significantly downregulated JAK2/STAT3 phosphorylation induced by IL-17. **(C)** IL-6 also recovered IL-17-stimulated JAK2/STAT3 phosphorylation in siRNA-AKT-Huh7 cells. **(D)** As shown by Matrigel invasion assay, STAT3-siRNA significantly reversed tumor invasion by IL-17 stimulation. IL-6 mAb (10 ng/ml for 36 h) completely reversed IL-17-induced HCC invasion, while IL-6 (100 ng/ml for 36 h) completely recovered IL-17-stimulated invasion of siRNA-AKT-Huh7 cells. Three separate experiments were performed in each study. Data are expressed as mean ± SD; Student's *t* test; # $p > 0.05$; *$p < 0.05$, **$p < 0.01$, and ***$p < 0.001$.

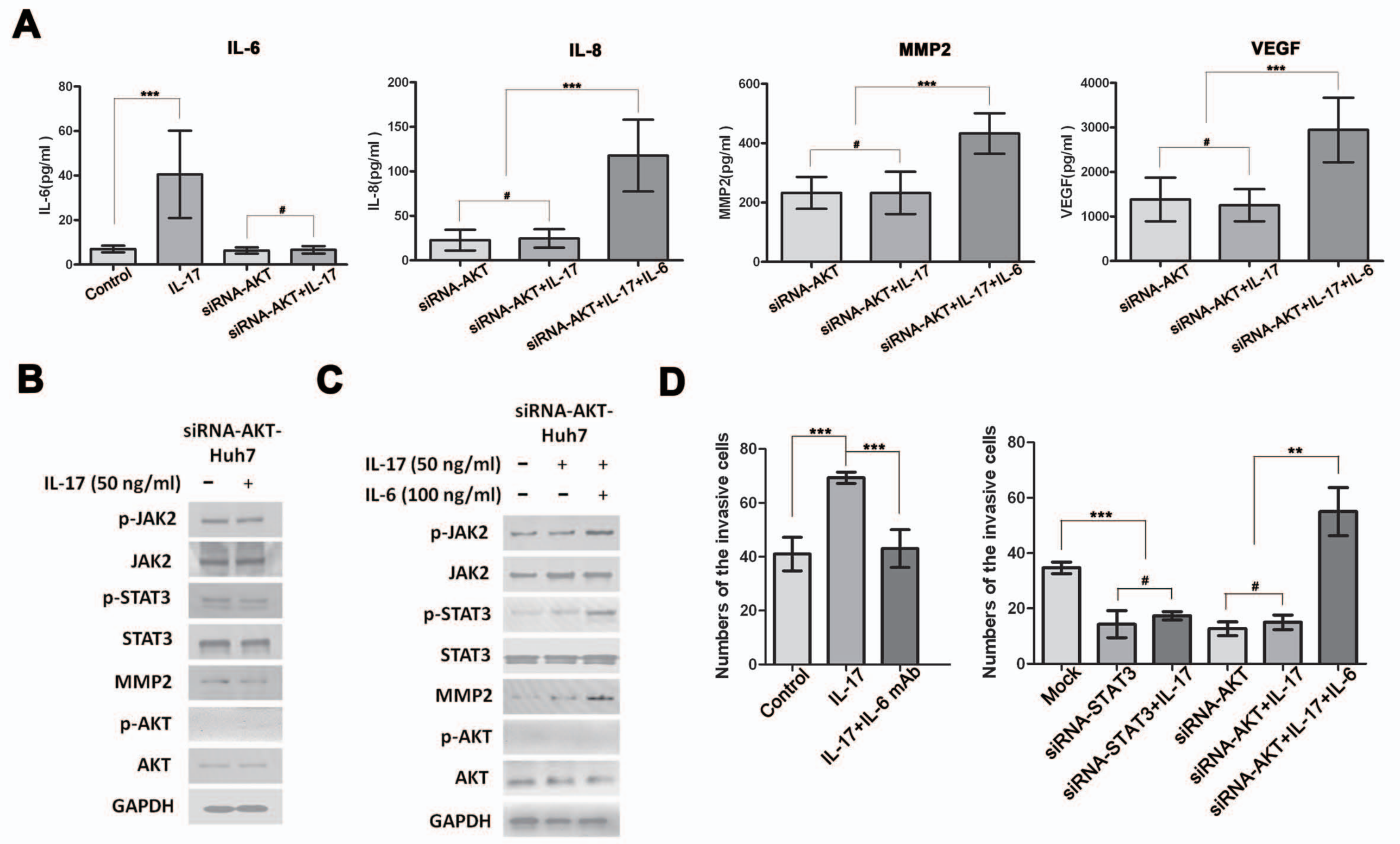
A
IL-6
IL-6(pg/ml)
Control
IL-17
siRNA-AKT
siRNA-AKT+IL-17
IL-8
IL-8(pg/ml)
siRNA-AKT
siRNA-AKT+IL-17
siRNA-AKT+IL-17+IL-6
MMP2
MMP2(pg/ml)
VEGF
VEGF(pg/ml)
B
siRNA-AKT-Huh7
IL-17 (50 ng/ml)
p-JAK2
JAK2
p-STAT3
STAT3
MMP2
p-AKT
AKT
GAPDH
C
siRNA-AKT-Huh7
IL-17 (50 ng/ml)
IL-6 (100 ng/ml)
D
Numbers of the invasive cells
Control
IL-17
IL-17+IL-6 mAb
Mock
siRNA-STAT3
siRNA-STAT3+IL-17
siRNA-AKT
siRNA-AKT+IL-17
siRNA-AKT+IL-17+IL-6
