# Supplementary material for: IL-17 induces AKT-dependent IL-6/JAK2/STAT3 activation and tumor progression in hepatocellular carcinoma
Source: Mol Cancer. 2011 Dec 15;10:150. doi: 10.1186/1476-4598-10-150 (PMC3310750; doi:10.1186/1476-4598-10-150)

Additional file 5

Figure S5 IL-17 promotes tumor cell proliferation and inhibites apoptosis of HCC cells in nude mice. Proliferation index and apoptosis index in xenografts were assayed with Ki-67 and TUNEL staining (200× magnification). SMMC7721-IL-17-derived xenografts showed increased tumor cell proliferation while reduced apoptosis of HCC cells compared with the SMMC7721-mock group. Data are expressed as mean ± SD; Student's *t* test; *p < 0.05.

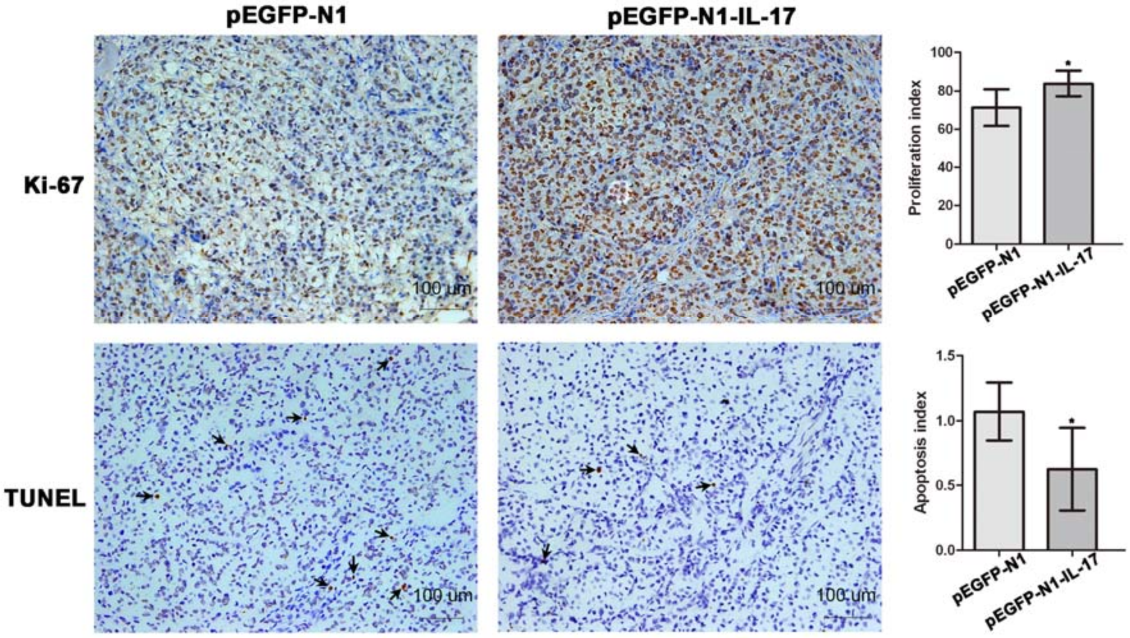

Supplement: Additional file 5 — Figure S5 IL-17 promotes tumor cell proliferation and inhibites apoptosis of HCC cells in nude mice. Proliferation index and apoptosis index in xenografts were assayed with Ki-67 and TUNEL staining (200× magnification). SMMC7721-IL-17-derived xenografts showed increased tumor cell proliferation while reduced apoptosis of HCC cells compared with the SMMC7721-mock group. [file 1476-4598-10-150-S5.PDF]
