# Supplementary material for: IL-17 induces AKT-dependent IL-6/JAK2/STAT3 activation and tumor progression in hepatocellular carcinoma
Source: Mol Cancer. 2011 Dec 15;10:150. doi: 10.1186/1476-4598-10-150 (PMC3310750; doi:10.1186/1476-4598-10-150)

**Additional file 6**

**Figure S6 The intratumoral p-STAT3 intensity is positively correlated with IL-17+ cell, neutrophil and microvessel densities.** Serial whole tumor sections from 87 HCC patients were used for immunostaining. The intratumoral p-STAT3 staining intensity was significantly and positively correlated with the levels of intratumoral IL-17+ cells, neutrophils, and MVD. Correlations between immunostaining parameters were analyzed by Spearman's rho coefficient test.

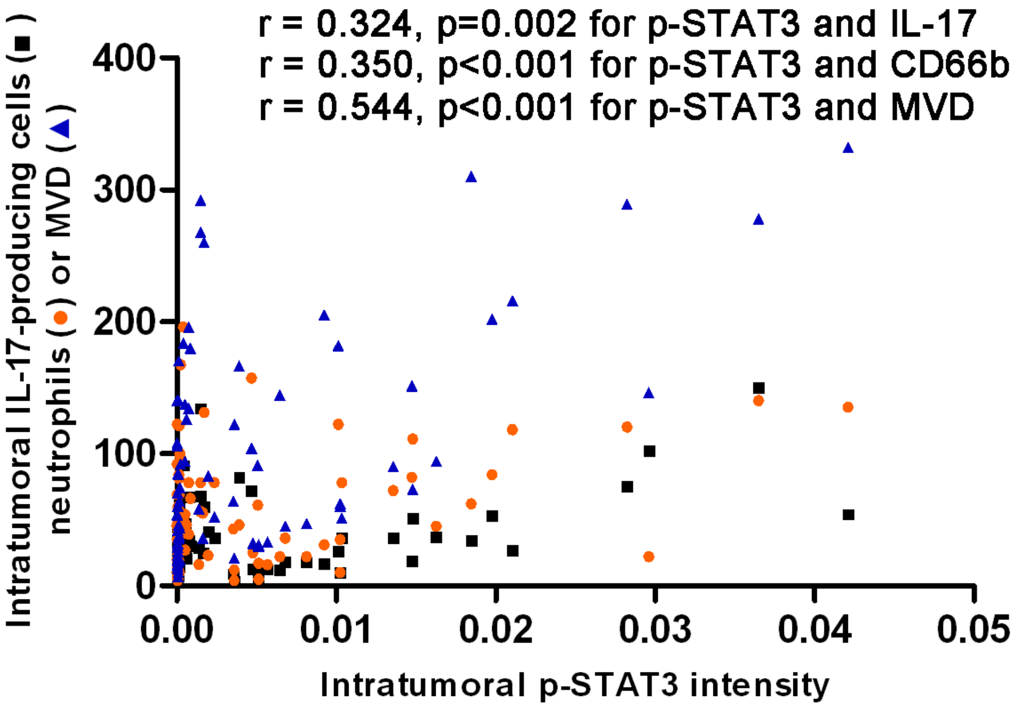

Supplement: Additional file 6 — Figure S6 The intratumoral p-STAT3 intensity is positively correlated with IL-17+ cell, neutrophil and microvessel densities. Serial whole tumor sections from 87 HCC patients were used for immunostaining. The intratumoral p-STAT3 staining intensity was significantly and positively correlated with the levels of intratumoral IL-17+ cells, neutrophils, and MVD. Correlations between immunostaining parameters were analyzed by Spearman's rho coefficient test. [file 1476-4598-10-150-S6.PDF]
