# Supplementary material for: IL-17 induces AKT-dependent IL-6/JAK2/STAT3 activation and tumor progression in hepatocellular carcinoma
Source: Mol Cancer. 2011 Dec 15;10:150. doi: 10.1186/1476-4598-10-150 (PMC3310750; doi:10.1186/1476-4598-10-150)

Additional file 7

Figure S7 Representative cases of immunohistochemical staining of IL-17 and p-STAT3 in a tissue microarray. Representative cases of immunohistochemical staining of IL-17 and p-STAT3 in a TMA are shown. Consecutive sections of case 1 and case 2 showed high IL-17+ cells and p-STAT3 staining, while consecutive sections of case 3 and 4 showed low IL-17+ cells and p-STAT3 staining. Magnification, 100× and 400×.

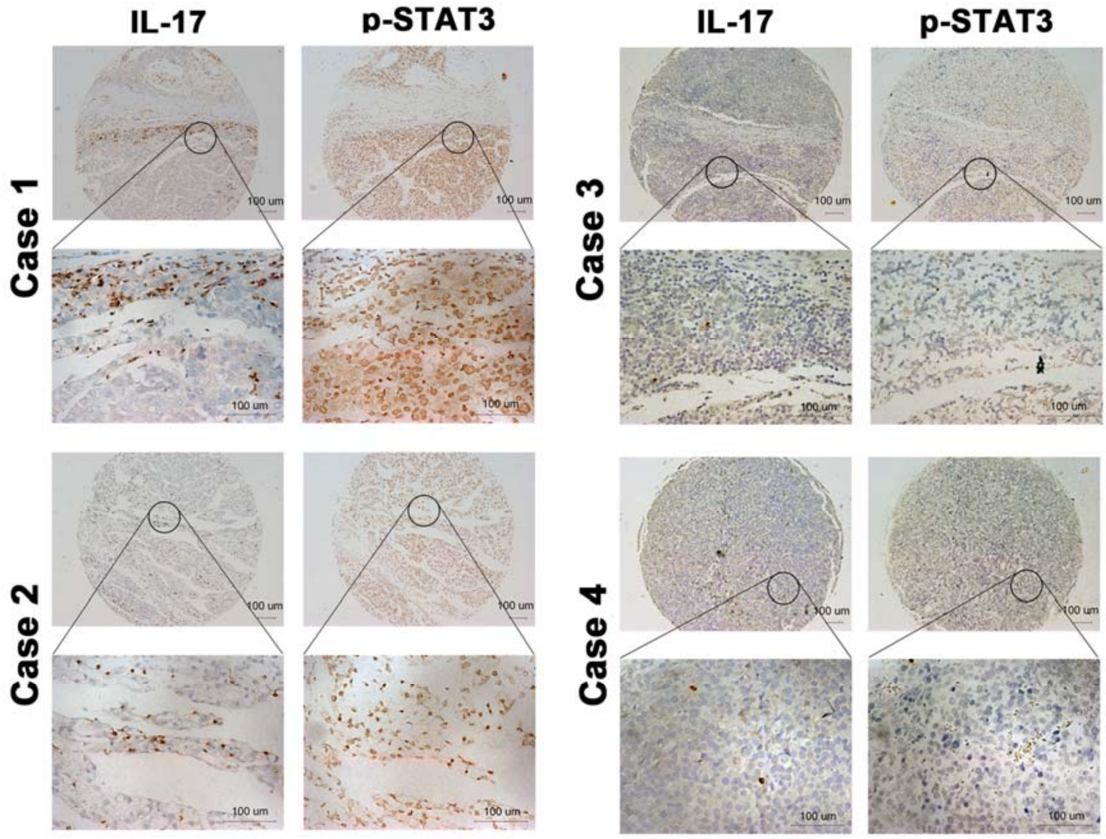

Supplement: Additional file 7 — Figure S7 Representative cases of immunohistochemical staining of IL-17 and p-STAT3 in a tissue microarray. Consecutive sections of case 1 and case 2 showed high IL-17+ cells and p-STAT3 staining, while consecutive sections of case 3 and 4 showed low IL-17+ cells and p-STAT3 staining. [file 1476-4598-10-150-S7.PDF]
