# Supplementary material for: IL-17 induces AKT-dependent IL-6/JAK2/STAT3 activation and tumor progression in hepatocellular carcinoma
Source: Mol Cancer. 2011 Dec 15;10:150. doi: 10.1186/1476-4598-10-150 (PMC3310750; doi:10.1186/1476-4598-10-150)
Supplement: Additional file 8 — Table S1 Correlation between IL-17 or p-STAT3 and clinicopathological characteristics. Both IL-17high and p-STAT3high were significantly correlated with microvascular invasion. [file 1476-4598-10-150-S8.PDF]

Additional file 8

Table S1：Correlation between IL-17 or p-STAT3 and clinicopathological characteristics

Variables	No. of Patients		*P*	No. of Patients		*P*
	IL-17 (Low)	IL-17 (High)		STAT3 (Low)	STAT3 (High)	
**Age, years**						
≤50	83	83	0.954	90	76	0.829
>50	78	79		87	70	
**Gender**						
female	27	19	0.195	30	16	0.125
male	134	143		147	130	
**HBsAg**						
no	14	31	0.010	27	18	0.450
yes	147	131		150	128	
**Liver cirrhosis**						
no	19	17	0.709	23	13	0.314
yes	142	145		158	129	
**alpha-Fetoprotein (ng/ml)**						
≤ 20	52	44	0.312	51	45	0.694
> 20	109	118		126	101	
**Tumor size (cm)**						
≤5	81	83	0.868	95	69	0.251
>5	80	79		82	77	
**Tumor differentiation**						
I/II	129	126	0.605	143	112	0.385
III/IV	32	36		34	34	
**Tumor number**						
Single	142	134	0.162	150	126	0.693
Multiple	19	28		27	20	
**Tumor encapsulation**						
None	86	78	0.344	97	67	0.111
Complete	75	84		80	79	
**Vascular invasion**						
No	98	77	0.016	107	68	0.013
yes	63	85		70	78	
**TNM stage**						
I	87	67	0.023	94	60	0.031
II-III	74	95		83	86	

Categorical variables were compared by $\chi2$ test.

Abbreviations: HBsAg, hepatitis B surface antigen; TNM, tumor-node-metastasis.
