# Supplementary material for: IL-17 induces AKT-dependent IL-6/JAK2/STAT3 activation and tumor progression in hepatocellular carcinoma
Source: Mol Cancer. 2011 Dec 15;10:150. doi: 10.1186/1476-4598-10-150 (PMC3310750; doi:10.1186/1476-4598-10-150)
Supplement: Additional file 9 — Table S2 Multivariate analyses of prognosis factors associated with survival. IL-17+ cell and p-STAT3 expression independently correlated with OS and recurrence, irrespective of being used alone or in combination. However, concomitant high of IL-17 and p-STAT3 was superior to either marker alone in terms of hazard ratios and p values for both OS and recurrence. [file 1476-4598-10-150-S9.PDF]

Additional file 9

Table S2：Multivariate analyses of prognosis factors associated with survival

Overall survival	A		B		C	
	HR (95% CI)	*P*	HR (95% CI)	*P*	HR (95% CI)	*P*
Gender (female vs male)	NA	NS	NA	NS	NA	NS
α-Fetoprotein (≤20 vs >20 ng/mL)	NA	NS	NA	NS	NA	NS
Tumor diameter (≤5 vs >5 cm)	2.279 (1.650- 3.148)	<0.001	2.182 (1.576 - 3.021)	<0.001	2.192 (1.583-3.035)	<0.001
Tumor number (single vs multiple)	NA	NS	NA	NS	NA	NS
Tumor encapsulation (none vs complete)	1.548 (1.126-2.128)	0.014	1.525 (1.110-2.095)	0.009	1.478 (1.073-2.034)	0.017
Tumor differentiation (I/II vs III/IV)	1.611 (1.139-2.280)	0.006	1.567 (1.107-2.218)	0.011	1.581 (1.118-2.237)	0.010
TNM stage (I vs II/III)	NA	NA	NA	NA	NA	NA
Vascular invasion (no vs yes)	1.967 (1.420-2.726)	<0.001	1. 957 (1.413-2.712)	<0.001	1.965 (1.413-2.732)	<0.001
IL-17 ^{low} vs IL-17 ^{high}	1.522 (1.112-2.082)	0.007				
p-STAT3 ^{low} vs p-STAT3 ^{high}			1.811 (1.324-2.478)	<0.001		
Combination of IL-17 and p-STAT3*						
Overall					NA	<0.001
I vs II					NA	NS
I vs III					2.244 (1.529-3.293)	<0.001

Cumulative Recurrence	A		B		C	
	HR (95% CI)	*P*	HR (95% CI)	*P*	HR (95% CI)	*P*
Gender (female vs male)	1.706 (1.044-2.786)	0.025	1.733 (1.061-2.829)	0.028	1.765 (1.078-2.891)	0.024
Tumor diameter (≤5 vs >5 cm)	1.645 (1.209 -2.240)	<0.001	1.610 (1.181-2.195)	0.003	1.613 (1.183-2.200)	0.003
Tumor encapsulation (none vs complete)	1.564(1.151-2.126)	0.005	1.567 (1.154-2.128)	0.004	1.532 (1.126-2.084)	0.007
TNM stage (I vs II/III)	NA	NA	NA	NA	NA	NA
Vascular invasion (no vs yes)	1.551 (1.132-2.126)	0.006	1.587 (1.157-2.175)	0.004	1.591 (1.157-2.188)	0.004
IL-17 ^{low} vs IL-17 ^{high}	1.360 (1.008 -1.836)	0.044				
p-STAT3 ^{low} vs p-STAT3 ^{high}			1.435 (1.064-1.937)	0.018		
Combination of IL-17 and p-STAT3*						
Overall					NA	0.009
I vs II					NA	NS
I vs III					1.718 (1.193-2.473)	0.004

Note: Univariate analysis was calculated using the Kaplan–Meier method (the log-rank test). Multivariate analysis was performed using the Cox multivariate proportional hazard regression model with a stepwise method (forward, likelihood ratio).

Abbreviations: HR, hazard ratio; CI, confidence interval; HBsAg, hepatitis B surface antigen; NA, not assessed; NS, not significant; TNM, tumor-node-metastasis.

* I, IL17 ^{high} and p-STAT3 ^{high}; II, IL17 ^{high} p-STAT3 ^{low} or IL17 ^{low} p-STAT3 ^{high}; III, IL17 ^{low} and p-STAT3 ^{low}.
